# Spliceosomal component PRP-40 is a central regulator of microexon splicing

**DOI:** 10.1016/j.celrep.2021.109464

**Published:** 2021-08-03

**Authors:** Bikash Choudhary, Olivia Marx, Adam D. Norris

**Affiliations:** 1 Biological Sciences, Southern Methodist University, Dallas, TX 75275, USA; 2 Lead contact

## Abstract

Microexons (≤27 nt) play critical roles in nervous system development and function but create unique challenges for the splicing machinery. The mechanisms of microexon regulation are therefore of great interest. We performed a genetic screen for alternative splicing regulators in the *C. elegans* nervous system and identify PRP-40, a core component of the U1 snRNP. RNA-seq reveals that PRP-40 is required for inclusion of alternatively spliced, but not constitutively spliced, exons. PRP-40 is particularly required for inclusion of neuronal microexons, and our data indicate that PRP-40 is a central regulator of microexon splicing. Microexons can be relieved from PRP-40 dependence by artificially increasing exon size or reducing flanking intron size, indicating that PRP-40 is specifically required for microexons surrounded by conventionally sized introns. Knockdown of the orthologous PRPF40A in mouse neuroblastoma cells causes widespread dysregulation of microexons but not conventionally sized exons. PRP-40 regulation of neuronal microexons is therefore a widely conserved phenomenon.

## In brief

Microexons play crucial roles in neuronal function but pose mechanistic challenges to the spliceosome. Choudhary et al. reveal that PRP-40, a core spliceosomal component, is required for microexon inclusion in *C. elegans* and mammalian cells. PRP-40 is proposed as a mediator of intron definition for exons refractory to exon definition.

## Graphical Abstract

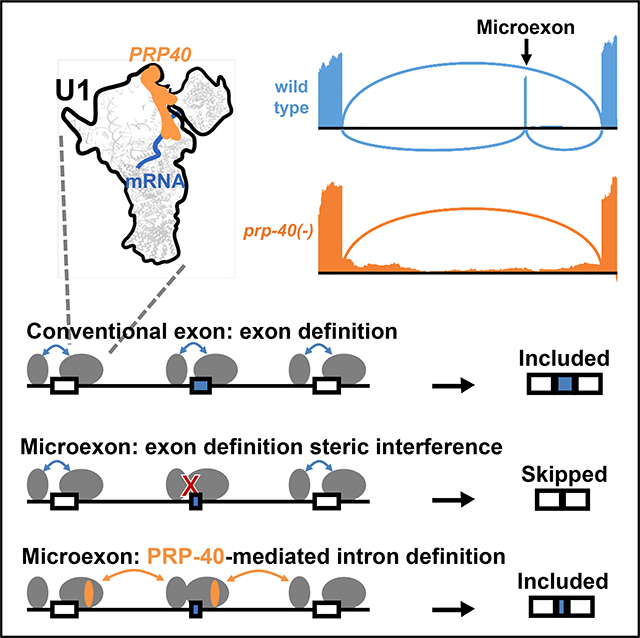

## INTRODUCTION

Small exons present unique challenges to the splicing machinery. Exons smaller than 51 nt are spliced with reduced efficiency *in vitro* and are often skipped *in vivo* (Dominski and Kole, 1991, 1992; Irimia et al., 2014). It has been hypothesized that these small exons fall below the detection limit for conventional “exon definition” models of splicing, in which the spliceosome initially recognizes and assembles across an exon (Berget, 1995; Robberson et al., 1990). Despite these mechanistic difficulties, genome-wide analyses reveal that microexons, often defined as exons of ≤27 nt, are enriched in neuronal genes, are frequently alternatively spliced, and are preferentially included in the brain compared with other tissues (Irimia et al., 2014; Li et al., 2015; Volfovsky et al., 2003). Microexons therefore define a network of alternatively spliced genes implicated in nervous system function, development, and disease (Gonatopoulos-Pournatzis and Blencowe, 2020; Ustianenko et al., 2017).

A number of sequence-specific RNA binding proteins have recently been identified that affect alternative splicing of subsets of microexons (Gonatopoulos-Pournatzis et al., 2018; Irimia et al., 2014; Li et al., 2015; Torres-Méndez et al., 2019), but the precise mechanisms by which these factors affect microexon recognition by the spliceosome remain under investigation (Gonatopoulos-Pournatzis et al., 2018). Traditionally, sequence-specific RNA binding proteins are thought to regulate splicing by binding a target pre-mRNA and either inhibiting or enhancing spliceosomal assembly, thus affecting the splicing outcome (Fu and Ares, 2014).

Recently, core components of the spliceosome itself have been shown to exert regulatory effects on specific types of alternative splicing (Dvinge et al., 2019; Papasaikas et al., 2015; Park et al., 2004; Saltzman et al., 2011). For example, knockdown of spliceosomal U1 snRNA preferentially leads to changes in 5′ splice site selection, while knockdown of U4 or U6 snRNA preferentially leads to intron retention (Dvinge et al., 2019). In principle, microexons could likewise constitute a class of splicing event regulated by specific spliceosomal components. Identification of such a component would yield important insight into the mechanisms by which sequence-specific RNA binding proteins and spliceosomal components coordinate microexon splicing.

We performed an *in vivo* genetic screen in the *C. elegans* nervous system for regulators of cell-specific splicing of *unc-16/JIP3*. We isolated alleles of *prp-40*, a component of the spliceosomal U1 snRNP. *prp-40* genetically and physically interacts with the neuronal sequence-specific RNA binding protein *exc-7/ELAV* to mediate neuronal inclusion of the *unc-16* alternative exon. Transcriptome-wide analysis revealed that PRP-40 is required for inclusion of alternatively spliced, but not constitutively spliced, exons. PRP-40 is particularly required for inclusion of small exons: the magnitude of splicing change upon PRP-40 loss is strongly dependent on exon size, and in the absence of PRP-40, the expression of nearly every microexon is reduced to essentially undetectable levels. Therefore, we consider PRP-40 a central regulator of microexon splicing. Microexons can be released from their dependence on PRP-40 by increasing their size or by reducing a flanking intron to unusually short lengths. Our data suggest a model in which microexons, too small for spliceosomal exon definition, can be recognized with the assistance of PRP-40 acting in a flanking intron through an intron definition mechanism. Finally, knockdown of the orthologous PRPF40A in mouse neuroblastoma cells causes widespread dysregulation of microexons but not conventionally sized exons, suggesting that the role of PRP-40 as a central regulator of microexon splicing is broadly conserved.

## RESULTS

### Spliceosomal component PRP-40 is required for cell-specific alternative splicing of *unc-16*/JIP3

**Figure 1.**
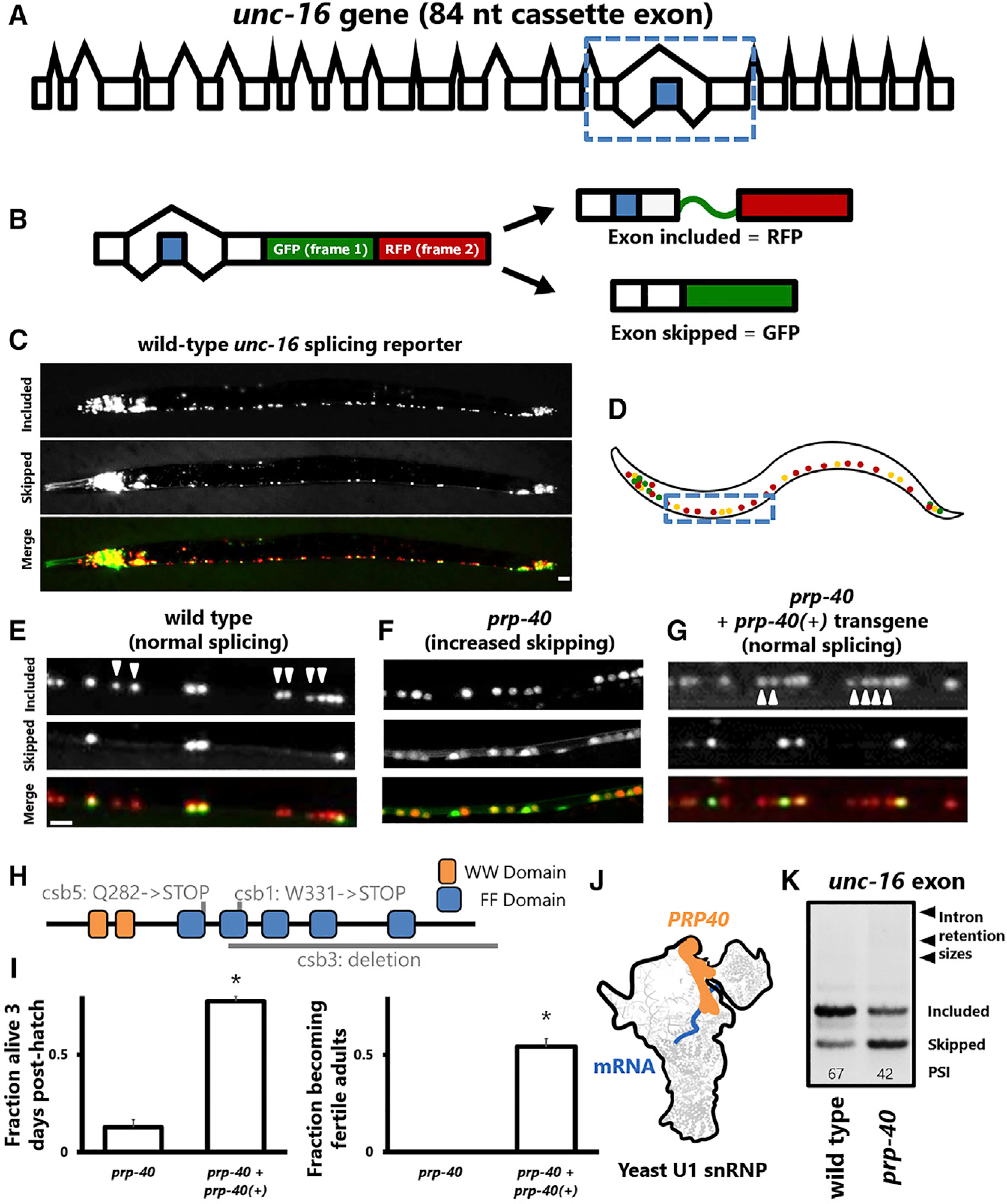
Spliceosomal component PRP-40 is required for cell-specific alternative splicing of *unc-16*/JIP3 (A) Gene model for *unc-16*, with alternative cassette exon highlighted in blue and dotted box indicating region cloned for minigene reporter. Not to scale (introns shortened). (B) Two-color fluorescent splicing reporter. Cassette exon (in blue) is engineered to encode a +1 nt frameshift, so that exon skipping will produce an in-frame GFP followed by a stop codon, but exon inclusion will shift GFP out of frame and without stop codons, followed by RFP translation in frame. (C) Whole-animal visualization of *unc-16* splicing reporter in the nervous system. Splicing pattern is invariant between wild-type animals (n > 20). Scale bar, 50 μm. (D) Schematic of the splicing reporter across the entire nervous system. Dotted box indicates region of the ventral nerve cord examined in (E)–(G). (E–G) Motor neurons of the ventral nerve cord in wild-type, *prp-40(−)*, and *prp-40(−)* + full-length *prp-40* fosmid rescuing transgene. In wild-type conditions, excitatory neurons express only the included isoform, but in *prp-40(−)* they express both isoforms. Splicing patterns are invariant among individuals of a given genotype (n ≥ 10 biological replicates). Arrowheads denote excitatory motor neurons. Scale bar, 10 μm. (H) Domain structure of *C. elegans* PRP-40 including the conserved WW and FF domains, overlaid with the locations of mutations identified in the genetic screen. *csb3* deletion allele is used in all following experiments. (I) *prp-40* transgene rescues lethality and sterility of *prp-40(−)*. Differences are statistically significant (unpaired two-tailed t test, p < 0.01). (J) Cartoon representation of the location of PRP-40 within the yeast U1 snRNP, as determined by cryo-EM (Li et al., 2019). (K) RT-PCR on whole-animal endogenous *unc-16* RNA confirms that *prp-40(−)* causes partial loss of *unc-16* exon inclusion. See also Figure S1.

To identify cell type-specific regulators of alternative splicing, we performed a forward genetic screen in *C. elegans* using an *in vivo* fluorescent splicing reporter. The two-color reporter is designed to visualize cassette exons, which can either be included or skipped in the mature mRNA (Figures 1A and 1B). The reporter will produce RFP when the alternative exon is included and GFP when the exon is skipped (Figures 1A and 1B). A frameshift of +1 nt engineered into the alternative exon causes a translational reading frameshift upon exon inclusion to generate an in-frame RFP instead of an in-frame GFP (Figures 1A and 1B). Applying fluorescent splicing reporters to the transparent nematode *C. elegans* enables visualization of tissue-specific and cell-specific splicing regulation *in vivo* (Gracida et al., 2016; Kuroyanagi et al., 2006; Thompson et al., 2019).

We previously identified an 84 nt cassette exon in the neuronal *unc-16*/*JIP3* kinase gene that exhibits different splicing patterns in different neuron types (Norris et al., 2014) (Figures 1A–1D). For example, the exon is fully included in excitatory motor neurons but is partially skipped in inhibitory motor neurons (Figures 1C–1E). Unbiased forward genetic screens identified a pair of sequence-specific RNA binding proteins, *unc-75/CELF* and *exc-7/ELAV*, which coordinately establish this neuron subtype-specific splicing (Norris et al., 2014). In this screen, we isolated additional alleles that affect *unc-16* splicing but were unable to determine their identities because they resulted in lethality during larval development. In each of the mutant strains, excitatory motor neurons lost their pattern of full exon inclusion and instead adopted a partially included splicing phenotype (Figure 1F; Figures S1A–S1C).

In this present study, we performed whole-genome sequencing of individual homozygous mutant larvae prior to lethality using whole-genome amplification and sequencing technology (Spits et al., 2006), which revealed that each of the mutant strains harbors a unique mutation in the gene *ZK1098.1* (Figures 1F–1H). *ZK1098.1* is the worm ortholog of yeast PRP40 and mammalian PRPF40A (Arribere et al., 2020), and henceforth we refer to it as *prp-40*. Transgenic overexpression of *C. elegans* PRP-40 using the *prp-40* promoter rescues both the lethality and the cell-specific splicing defects of a *prp-40* mutant (Figures 1G–1I).

PRP-40 orthologs in yeast and humans are constituents of the spliceosomal U1 snRNP (Becerra et al., 2016; Kao and Siliciano, 1996; Li et al., 2019) (Figure 1J). In *S. cerevisiae*, PRP40 is an essential gene required for constitutive splicing of the few introns that were examined (Kao and Siliciano, 1996). In contrast, we find that *C. elegans prp-40* mutants appear to affect *unc-16* alternative splicing but not constitutive splicing. First, the splicing reporter does not exhibit reduced fluorescence (which would indicate a failure to excise stop-codon encoding introns) in *prp-40* mutants, but rather a shift from RFP to GFP (indicating a change in isoform use). Second, RT-PCR confirms that the *unc-16* alternative exon undergoes increased skipping in *prp-40* mutants but does not undergo increased intron retention (Figure 1K). In sum, we have identified a component of the U1 snRNP that affects alternative, but not constitutive, splicing of *unc-16* in neurons.

### *prp-40* genetically interacts with RNA binding protein *exc-7/*ELAV to control neuronal splicing of *unc-16*

**Figure 2.**
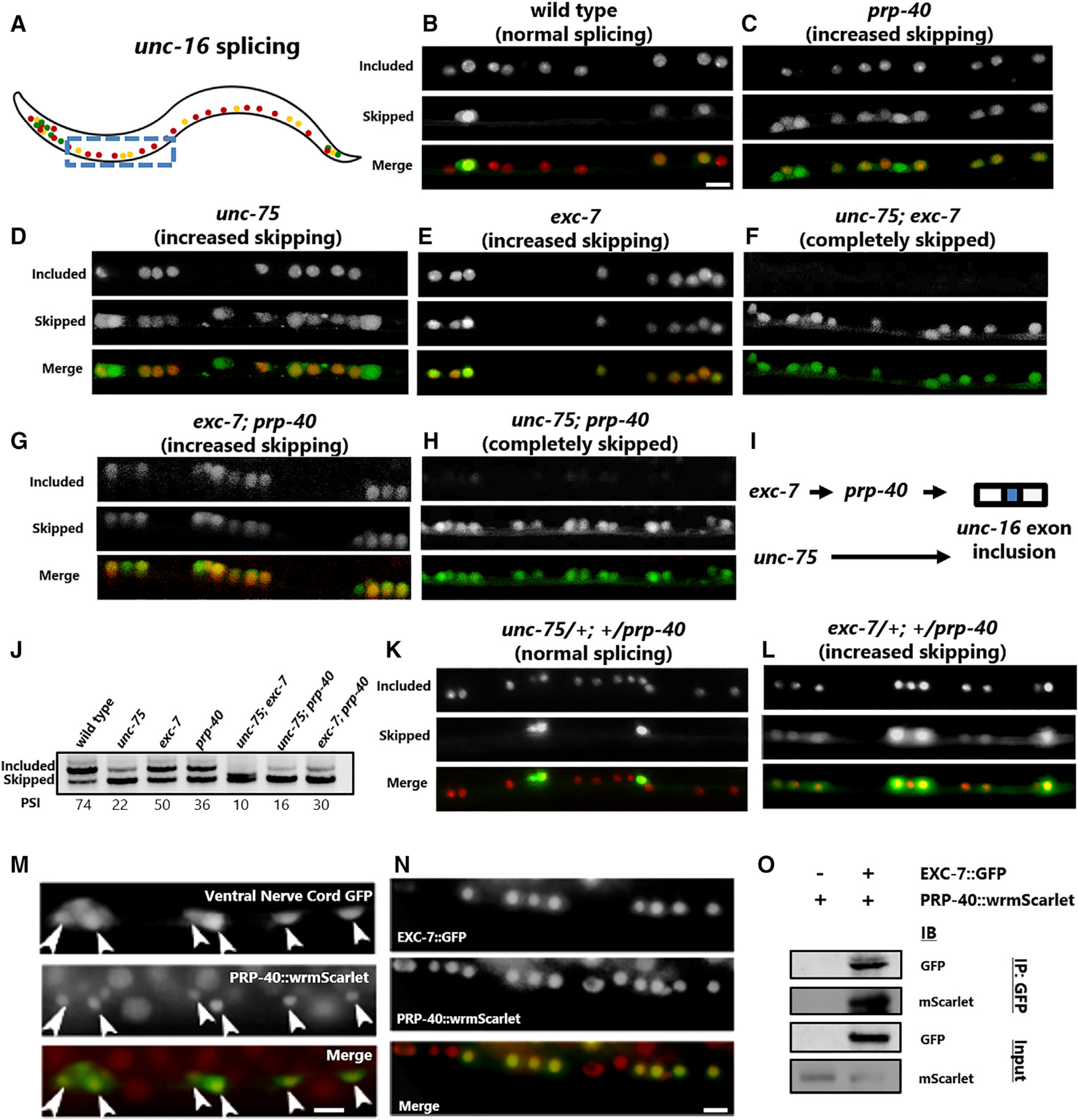
PRP-40 genetically interacts with RNA binding protein *exc-7/*ELAV to control neuronal splicing of *unc-16* (A) Schematic of *unc-16* splicing reporter. Boxed region indicates nerve cord visualized throughout the figure. (B–L) *unc-16* splicing reporter in motor neurons of the ventral nerve cord. Note that cell identity and numbers do not change in *prp-40* mutants (see also Figures S2B and S2C). Scale bar, 10 μm. In wild-type (B), excitatory motor neurons express only the included isoform, while inhibitory motor neurons express both isoforms. In either *unc-75* (D) or *exc-7* (E) RNA binding protein mutants, excitatory motor neurons express both isoforms. *prp-40* (C) mutants likewise express both isoforms in excitatory motor neurons (as negative control, same image as Figure 1F). Both *unc-75; exc-7* (F) and *unc-75; prp-40* (G) double mutants completely lose exon inclusion in all neurons, while *exc-7; prp-40* (H) double mutants resemble either single mutant. (I) Genetic model of *unc-16* splicing showing *prp-40* in same genetic pathway as *exc-7*, parallel to *unc-75*. (L) Whole animal RT-PCR mirrors the results from cell-specific splicing reporters. (K) *exc-7/+; +/prp-40* resemble either homozygous single mutant, while (L) *unc-75/+; +/prp-40* trans-heterozygotes resemble wild-type. Splicing reporter phenotypes are invariant across individuals of a given genotype (n ≥ 7). (M) Pan-neuronal GFP co-labels PRP-40-positive motor neurons in the ventral nerve cord. (N) Endogenously labeled EXC-7::GFP is co-expressed with endogenously labeled PRP-40 wrmScarlet in motor neurons of the ventral nerve cord. (O) PRP-40::wrmScarlet co-immunoprecipitates with EXC-7::GFP immunoprecipitated with α-GFP antibody, but not under control (un-tagged EXC-7) conditions. See also Figure S2.

We previously identified a pair of sequence-specific RNA binding proteins, *unc-75/CELF* and *exc-7/ELAV*, that coordinately control *unc-16* splicing in different neuron types (Norris et al., 2014). When both *unc-75* and *exc-7* are lost, the alternative exon is completely skipped throughout the nervous system. However, when only one RNA binding protein is lost, the splicing phenotype resembles a *prp-40* mutant (Figures 2A–2E; Figure S2A). For example, in the excitatory motor neurons, the exon goes from being fully included to partially included (Figures 2B–2E). Therefore, we wanted to test whether *prp-40* genetically interacts with *exc-7* and/or *unc-75*.

*exc-7; prp-40* double mutants are similar to either *exc-7* or *prp-40* single mutants, exhibiting partial exon inclusion in motor neurons throughout the nerve cord (Figure 2G). On the other hand, *unc-75; prp-40* mutants exhibit a strong genetic interaction, resulting in complete loss of exon inclusion throughout the nervous system, similar to *unc-75; exc-7* double mutants (Figures 2F–2H). These genetic data are consistent with PRP-40’s functioning in a linear pathway with *exc-7*, but in a parallel pathway to *unc-75*, to control *unc-16* alternative splicing in motor neurons (Figure 2I). RT-PCR from whole worms, although lacking the cell-specific resolution of the *in vivo* reporters, further confirmed the genetic results (Figure 2J).

To further test this genetic model, we generated trans-heterozygous animals. *unc-75/+; +/prp-40* mutants are indistinguishable from wild-type animals, but *exc-7/+; +/prp-40* mutants resemble homozygous *exc-7* or *prp-40* single mutants (Figures 2K and 2L). Such “non-allelic non-complementation” often indicates that two gene products function together through physical interaction and/or as part of the same molecular pathway (Yook, 2005).

These results indicate that *prp-40* functions in a linear genetic pathway with *exc-7* but not *unc-75.* We previously found that EXC-7 binds to specific *cis*-elements located in the downstream intron of *unc-16* to regulate exon inclusion (Norris et al., 2014). Coupled with our genetic results, this suggests the hypothesis that *exc-7* could bind to the *unc-16* intron and recruit spliceosomal component PRP-40 to mediate exon recognition.

### PRP-40 and EXC-7 physically associate

We generated an endogenously tagged *PRP-40::wrmScarlet* translational reporter and detected widespread expression throughout development and in many tissues, including the nervous system, intestine, muscle, and gonad (Figures S2D and S2E). We detected PRP-40 expression in the motor neurons of the ventral nerve cord, where PRP-40 is required for regulation of *unc-16* splicing (Figure 2M). Expression is exclusively nuclear, consistent with its role as a spliceosomal component (Figure S2E).

We next examined whether PRP-40 is co-expressed with EXC-7 using an endogenously tagged EXC-7::GFP strain (Pham and Hobert, 2019). As previously reported, EXC-7 is expressed in multiple cell types (Pham and Hobert, 2019), including the excitatory motor neurons of the ventral nerve cord, where, like PRP-40, it is localized to the nucleus (Figure 2N). Within individual cells, we found that both factors are present in the nucleus and are excluded from the nucleolus, and observed multiple puncta containing both *PRP-40::wrmScarlet* and *EXC-7::GFP* (Figures S2F and S2G).

Finally, we performed co-immunoprecipitation experiments to determine whether EXC-7 and PRP-40 physically interact. Immunoprecipitation of *EXC-7::GFP* with an α-GFP antibody yielded robust co-immunoprecipitation of *PRP-40::wrmScarlet* (Figure 2O). This interaction was specific, as co-immunoprecipitation was not observed with un-tagged EXC-7 (Figure 2O). These results demonstrate that, in addition to their genetic interactions, EXC-7 and PRP-40 physically interact.

### PRP-40 regulates alternative splicing, but not constitutive splicing, transcriptome-wide

**Figure 3.**
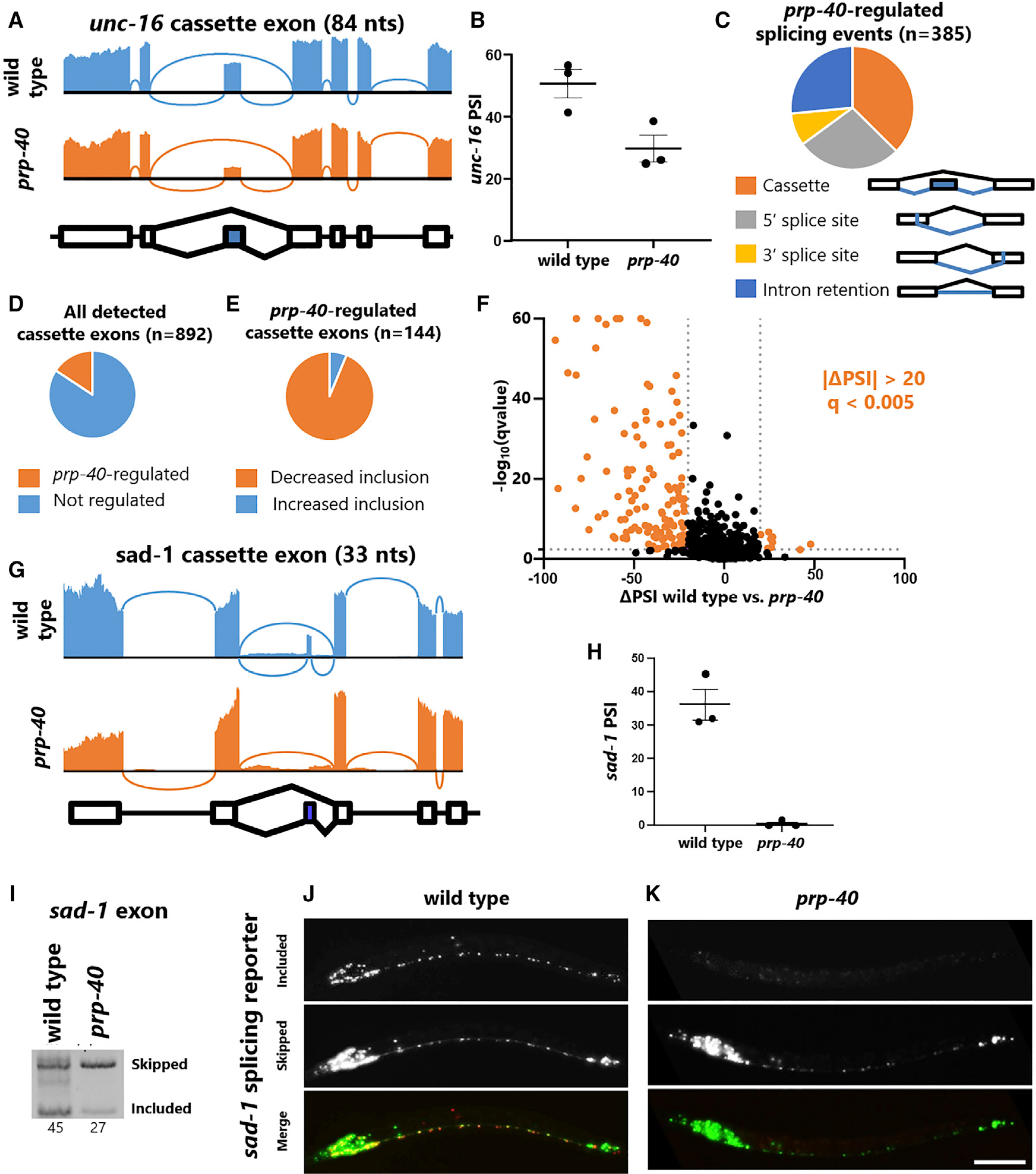
PRP-40 regulates alternative splicing, but not constitutive splicing, transcriptome-wide (A) Sashimi plot of RNA-seq data confirming *unc-16* cassette exon undergoes loss of inclusion in *prp-40(−)*, but flanking introns and exons are spliced normally. (B) Percentage spliced in (PSI) values for the *unc-16* cassette exon obtained from RNA-seq triplicates. (C) All significant (|ΔPSI| ≥ 20, q < 0.01) splicing differences between wild-type and *prp-40(−)*, grouped by type of event. Cassette exons are the most dysregulated type of splicing event. (D) Among all detected cassette exons (at least five junction spanning reads for each isoform), PRP-40 regulates 16%. (E) Among PRP-40-regulated cassette exons (|ΔPSI| ≥ 20, q < 0.01), the majority (94%) exhibit decreased inclusion in the absence of PRP-40. (F) Volcano plot of all cassette exons detected. Colored data points indicate |ΔPSI| ≥ 20 and q < 0.005. (G) Sashimi plot of *sad-1* 33 nt cassette exon showing complete loss of exon inclusion in *prp-40(−)*. (H) PSI values for the *sad-1* cassette exon obtained from RNA-seq triplicates. (I) *sad-1* RT-PCR further confirms that exon inclusion is strongly downregulated in *prp-40(−)*. Internal primer within cassette exon was designed to yield a smaller product for the included isoform compared with the skipped isoform. (J and K) *sad-1 in vivo* splicing reporters confirm that the cassette exon is partially included in the wild-type nervous system, but completely skipped in *prp-40(−)*. Splicing phenotype is invariant within individuals of the same genotype (n ≥ 10 biological replicates). Scale bar, 50 μm. See also Figure S3.

To determine whether PRP-40 regulates alternative splicing across the transcriptome, we performed RNA sequencing (RNA-seq) on L3-staged animals, prior to the onset of lethality in *prp-40* mutants. RNA-seq confirmed loss of *unc-16* exon inclusion in *prp-40* mutants and further confirmed that splicing of flanking *unc-16* introns and exons are unaffected in *prp-40* mutants (Figures 3A and 3B). Therefore, the role of PRP-40 in regulating *unc-16* splicing is restricted to the *unc-16* alternative exon.

Transcriptome-wide analysis showed that PRP-40 regulates many types of alternative splicing, with cassette exons being the most common (Figure 3C). Among all alternative splicing events identified in our analysis, PRP-40 regulates only a small percentage of cassette exons (16%) (Figure 3D), and an even smaller percentage of other types of alternative splicing (Figures S3A and S3B; Table S1). The vast majority of PRP-40-regulated cassette exons exhibit decreased inclusion upon loss of PRP-40 (94%) (Figure 3E). In contrast with *unc-16*, whose cassette exon exhibits only ~20% reduction in inclusion, many cassette exons are strongly affected by loss of PRP-40 (91 cassette exons with >30% ΔPSI) (Figure 3F).

As an additional validation of our RNA-seq results, we visualized *in vivo* splicing of a small (33 nt) cassette exon in the *sad-1* kinase using a fluorescent reporter we previously generated (Thompson et al., 2019). RNA-seq indicates that the exon is ~35% included in wild-type animals, but completely skipped in *prp-40* mutants (Figures 3G and 3H). The *in vivo* reporter yields similar results: many neurons express both isoforms in wild-type animals, but in *prp-40* mutants, only the skipped isoform is expressed (Figures 3J and 3K; Figure S3C). RT-PCR analysis further confirms the loss of *sad-1* exon inclusion in *prp-40* mutants, with no appreciable change in retention of the flanking introns (Figure 3I). This agrees with RT-PCR and RNA-seq data indicating that *prp-40* mutants affect alternative exons but do not disrupt the splicing of neighboring constitutive exons (Figures 1K, 2A, and 2G). Together these results demonstrate that the spliceosomal component PRP-40 regulates a subset of alternative exons, with a particular role in facilitating cassette exon inclusion, but has little effect on constitutive splicing.

### PRP-40 is a central regulator of microexon splicing

**Figure 4.**
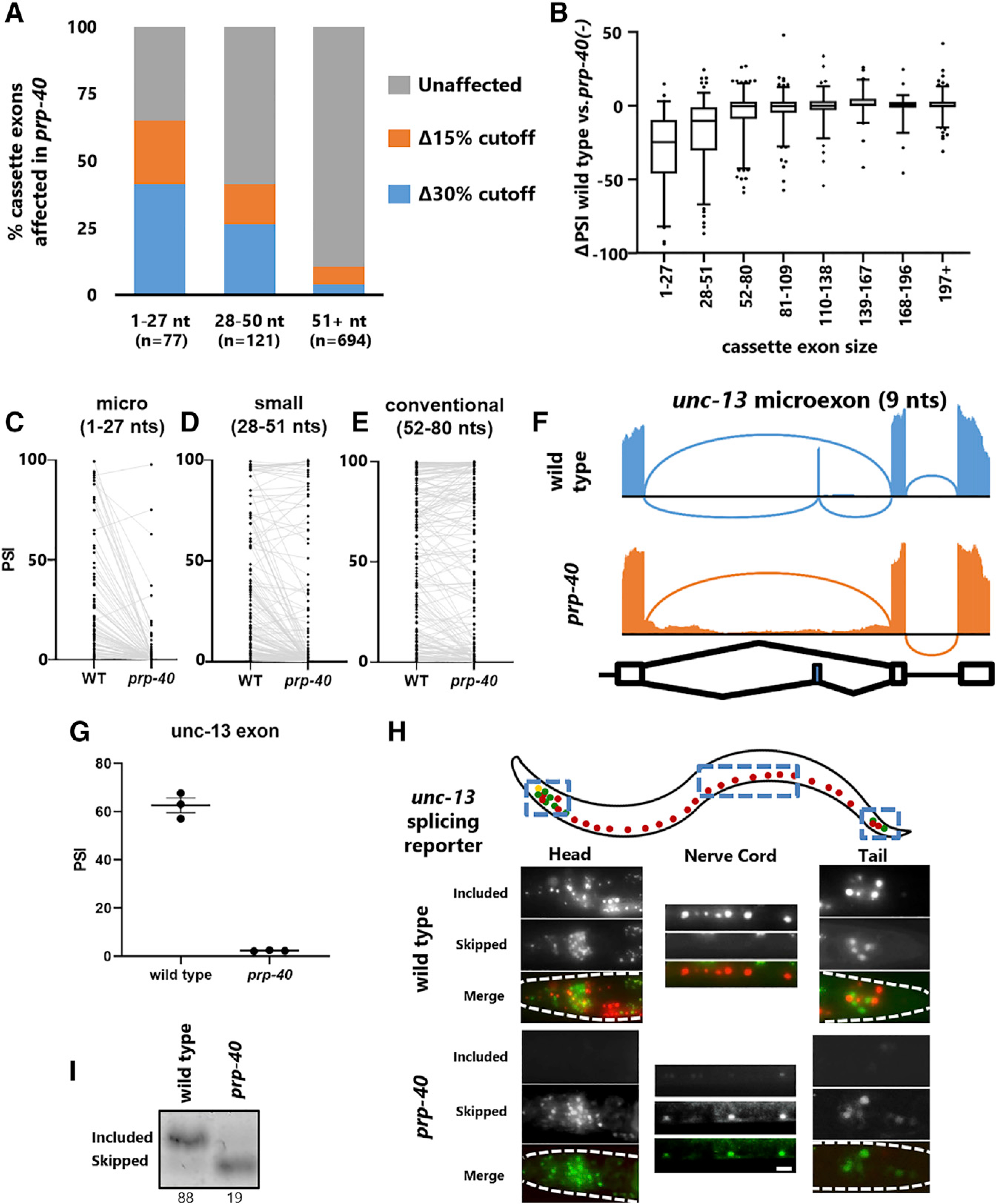
PRP-40 is a central regulator of microexon splicing (A) Percentage of cassette exons affected by *prp-40(−)* binned by size: 1–27 nt (microexons), 28–50 nt (small exons), and ≥51 nucleotides (conventionally sized exons), at either Δ15 or Δ30 PSI (q < 0.01). (B) Magnitude (ΔPSI) of change between wild-type and *prp-40(−)* for exons binned by exon size. Whiskers represent 5th and 95th percentiles. (C–E) PSI values for each exon in wild-type and in *prp-40(−)* for microexons (C), small exons (D), and conventional exons (E). (F) Sashimi plot showing complete loss of a 9 nt microexon in the *unc-13* gene in a *prp-40* mutant. (G) PSI values for the *unc-13* microexon obtained from RNA-seq triplicates. (H) *In vivo* splicing reporter for *unc-13* microexon shows that in wild-type animals, the microexon is included in many neurons, and skipped in many others, with few neurons expressing both isoforms simultaneously. In *prp-40(−)*, all neurons lose expression of the included isoform. Splicing patterns are invariant within a given genotype (n ≥ 7 biological replicates). Scale bar, 10 μm. (I) RT-PCR confirms that the *unc-13* microexon is lost in *prp-40(−)*. See also Figure S4.

We next searched for commonalities among the alternative exons regulated by PRP-40. One striking observation was that small exons are much more susceptible to loss of PRP-40 than are conventionally sized exons (Figure 4A). Although only <10% of conventionally sized exons (> 51 nt) are affected by loss of PRP-40, ~65% of microexons (defined here as 1–27 nt) and ~45% of small exons (28–51 nt) are affected (Figure 4A). The magnitude of splicing change in *prp-40* mutants is likewise strongly dependent on exon size, with microexons and small exons undergoing large reduction in exon inclusion (Figure 4B). This is exemplified by the small *sad-1* exon (33 nt) which is completely skipped in the absence of PRP-40, in comparison with the conventionally sized *unc-16* exon (84 nt), which is only partially skipped in the absence of PRP-40 (see Figures 3A and 3G).

Focusing on all detected microexons, we identified only two that are unaffected by loss of PRP-40 but included at appreciable levels (>20% included) in wild-type animals (Figure 4C). The remaining 97% of microexons are either strongly dependent on PRP-40 (>20% reduction) or not included at appreciable levels to begin with (<20% included in wild-type). This strong dependence on PRP-40 decreases as exon size increases (Figures 4D and 4E; Figures S4A–S4D). On the basis of these results, we propose PRP-40 as a central regulator of microexon splicing, given that (1) the vast majority of microexons require PRP-40 for inclusion, and (2) the requirement for PRP-40 is strongly dependent on exon size.

To further examine the role of PRP-40 as a microexon central regulator *in vivo*, we generated a splicing reporter for a 9 nt microexon in the synaptic regulatory gene *unc-13* (Madison et al., 2005). RNA-seq data revealed that both the skipped and included isoforms are expressed in wild-type animals (Figures 4F and 4G), and the *in vivo* reporter likewise revealed some neurons expressing the included isoform (RFP) and other neurons expressing the skipped isoform (GFP) (Figure 4H). Unlike reporters for *unc-16* or *sad-1*, in which many neurons express both isoforms, we noted that the wild-type *unc-13* splicing pattern is largely binary: most neurons express only one isoform or the other (Figure 4H). On the other hand, in *prp-40* mutants, exon inclusion is completely lost in all neurons (Figures 4H and 4I; Figure S4E). As in the case with *unc-16* and *sad-1*, exons and introns neighboring the *unc-13* microexon are unaffected in *prp-40* mutants (Figure 4F). Together these results identify PRP-40 as a central regulator of microexon splicing.

### PRP-40-regulated microexons define a network of neuronal transcripts

**Figure 5.**
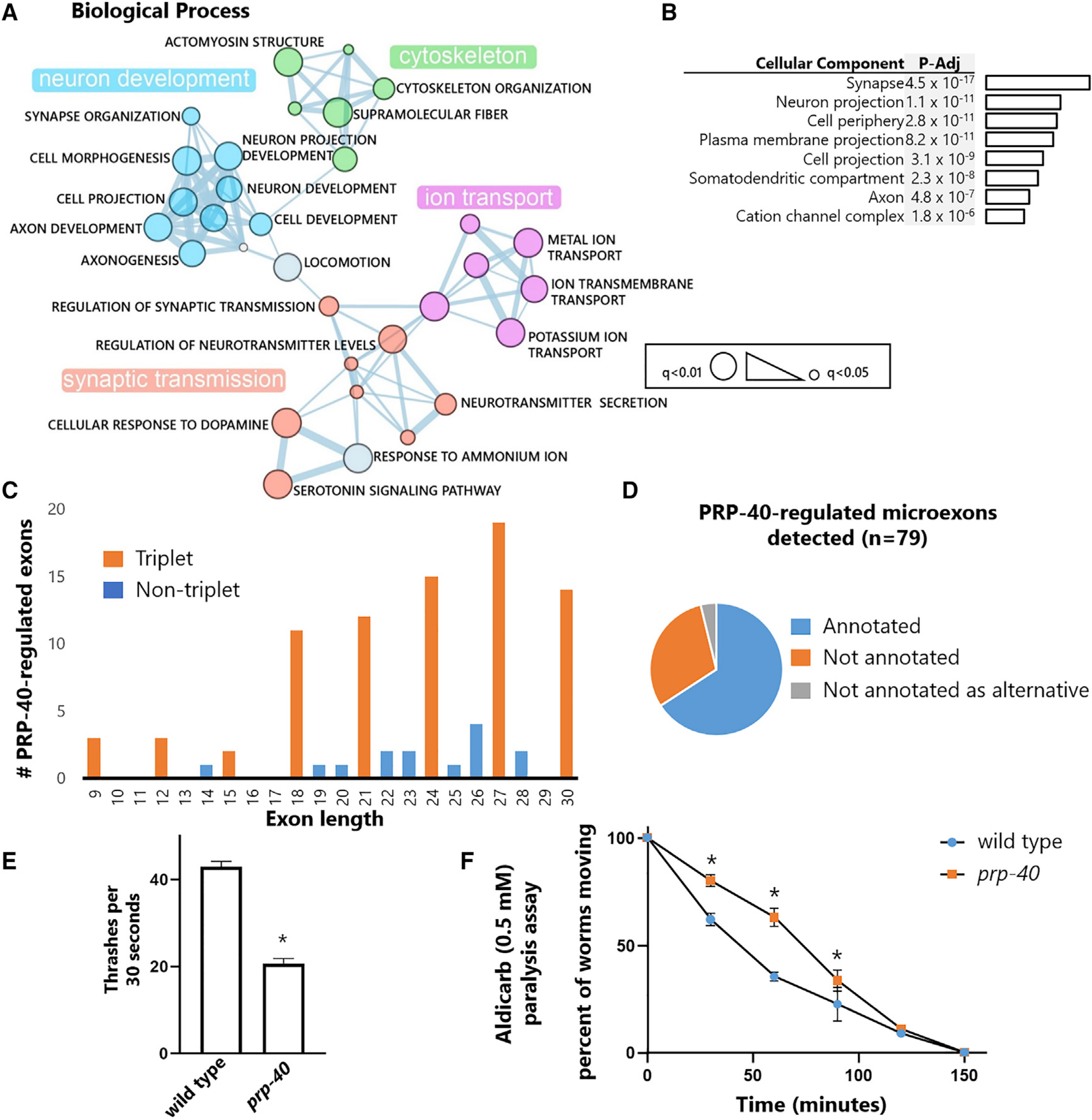
PRP-40-regulated microexons define a network of neuronal transcripts (A) Gene Ontology biological process analysis for genes containing PRP-40-regulated microexons (|ΔPSI| ≥ 20, q < 0.01) visualized by Enrichment Map plugin for Cytoscape. (B) Gene Ontology cellular component analysis on genes containing PRP-40*-*regulated microexons. (C) Histogram of PRP-40*-*regulated microexon size. Microexons with sizes divisible by 3 indicated in orange. (D) A substantial minority of PRP-40*-*regulated microexons < 30 nt (30%) are previously unannotated, and a small number (4%) are annotated as constitutive exons. (E) *prp-40(−)* L3-stage animals have defects in locomotion, as measured by rate of thrashing in liquid (M9 buffer). n = 20 animals per genotype. (F) *prp-40(−)* L3-stage animals are resistant to 0.2 mM aldicarb. n = 20 animals per genotype per replicate. Statistical test; paired t test with unequal variance and one way ANOVA test. Mean ± SEM (*p < 0.001).

To determine whether PRP-40-regulated microexons are present in specific functional gene classes, we performed Gene Ontology analysis on PRP-40-regulated microexons. We found strong neuronal functional enrichment in both biological processes (e.g., neuron development, synaptic transmission) and cellular components (e.g., synapse, neuron projection) (Figures 5A and 5B). These results are in line with studies of mammalian microexons that find strong functional enrichment for genes involved in neuronal development and disease (Gonatopoulos-Pournatzis and Blencowe, 2020; Scheckel and Darnell, 2015).

Mammalian microexons tend to be frame preserving (multiples of 3 nt), such that inclusion or skipping of the microexon does not cause translational frameshifts. We likewise find that PRP-40-regulated microexons show a strong preference for frame preservation (Figure 5C), in agreement with recent work on all detectable *C. elegans* microexons (Koterniak et al., 2020). We also tested whether the PRP-40-regulated microexons we identified are present in existing gene annotations and found that a substantial minority of microexons (30%) were previously unannotated, and a small number (4%) were previously annotated but considered constitutive exons (Figure 5D).

Given that PRP-40-regulated microexons show a strong functional enrichment for neuronal genes, we next asked whether *prp-40* mutant animals exhibit neuronal or behavioral defects. Indeed, we found that *prp-40* mutants exhibit a strong defect in locomotion (Figure 5E) as well as resistance to the acetylcholinesterase drug aldicarb (Figure 5F), which is indicative of a defect in synaptic transmission at the neuromuscular junction (Mahoney et al., 2006). Together these results demonstrate that PRP-40 regulates a network of neuronal, frame-preserving microexons, and is necessary for neuronal function.

### Molecular mechanisms underlying PRP-40-mediated microexon inclusion

**Figure 6.**
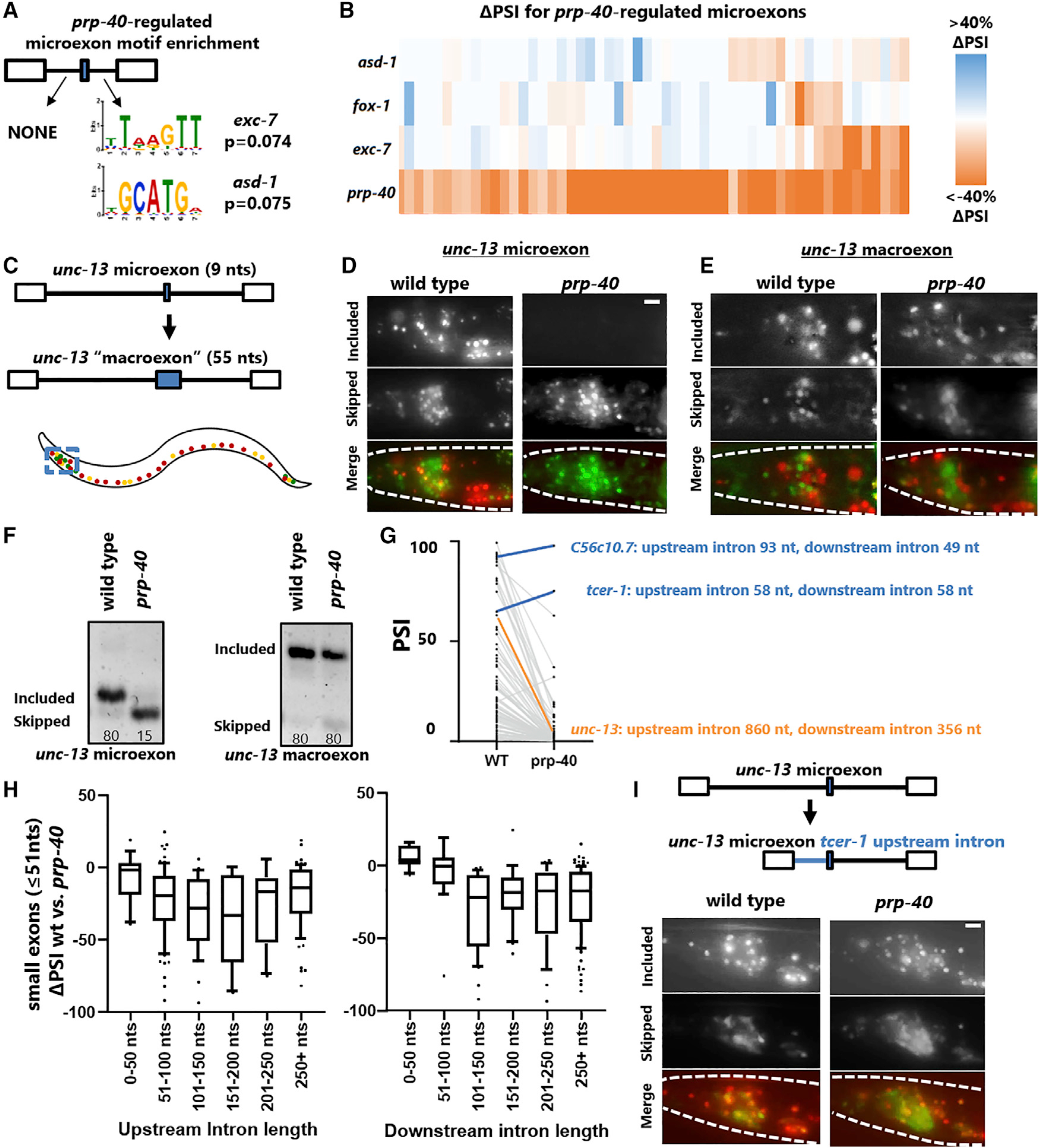
Molecular mechanisms underlying PRP-40-mediated microexon inclusion (A) Analysis of motif enrichment using MEME suite reveals no strongly enriched splicing factor *cis*-elements flanking PRP-40*-*regulated microexons but finds motifs (adjusted p = 0.07) for *exc-7* and *asd-1*. (B) RNA-seq analysis shows that *exc-7*, *fox-1*, and *asd-1* all regulate a small subset of PRP-40-regulated microexons, but no single splicing factor plays a dominant role. (C) *unc-13* microexon was expanded into a 55 nt “conventionally sized” exon. (D) Inclusion of the *unc-13* 9 nt microexon is completely dependent on PRP-40, as shown in more detail in Figure 4H. (E) Increasing the size of the *unc-13* microexon does not substantially alter its cell-specific splicing pattern but completely relieves its dependence on PRP-40. Splicing patterns are invariant among individuals of a given genotype (n ≥ 7). (F) RT-PCR confirms that the *unc-13* microexon, but not the engineered “macroexon,” is completely dependent on PRP-40. (G) Both exceptional microexons that are independent of PRP-40 have unusually short introns. (H) The magnitude of splicing dysregulation (ΔPSI) of small exons upon loss of PRP-40 depends on the size of the flanking introns, with shorter flanking introns resulting in less dependence on PRP-40. Whiskers represent 5th and 95th percentiles. (I) Replacing the long *unc-13* upstream intron with the short *tcer-1* upstream intron has minor effects on *unc-13* microexon splicing in wild-type animals but strongly relieves its dependence on PRP-40. Splicing patterns are invariant among individuals of a given genotype (n ≥ 5 biological replicates). Scale bars, 10 μm. See also Figure S5.

We next examined the mechanisms by which PRP-40 regulates microexons. Our experiments on *unc-16* splicing indicate that PRP-40 acts in concert with the sequence-specific neuronal splicing factor EXC-7/ELAV to mediate exon inclusion. We therefore asked whether PRP-40-regulated microexons are enriched for *cis*-elements corresponding to known sequence-specific splicing factors in their flanking introns. Analysis of motif enrichment using MEME suite (Bailey et al., 2009) did not identify any strong splicing factor *cis*-element enrichment but did identify weak (Bonferroni-corrected p = 0.07) enrichment for *exc-7* and *asd-1* binding motifs in downstream introns (Figure 6A).

To test whether EXC-7 or ASD-1 regulate PRP-40-dependent microexons, we analyzed existing RNA-seq datasets (Norris et al., 2017; Tan and Fraser, 2017) and found that *exc-7* and *asd-1* both regulate a small subset of PRP-40-regulated microexons (Figure 6B). We also performed RNA-seq on *fox-1* mutants, as *fox-1* and *asd-1* represent the worm counterparts of the mammalian Rbfox family of proteins, previously shown to mediate inclusion of a small subset of mammalian microexons (Gonatopoulos-Pournatzis et al., 2018; Li et al., 2015). We similarly find that FOX-1 controls a small subset of PRP-40-regulated microexons (Figure 6B). Together these results indicate that diverse sequence-specific RNA binding proteins co-regulate PRP-40-mediated microexon inclusion, with no single splicing factor playing a dominant role.

As the dependence on PRP-40 is strongly correlated with exon size, we wanted to test whether artificially increasing exon size is sufficient to modify a microexon’s dependence on PRP-40. We therefore expanded the *unc-13* microexon in our *in vivo* reporter from 9 to 55 nt (Figure 6C). We hoped that by simply repeating the wild-type nucleotide sequence of the *unc-13* microexon multiple times, we would avoid creating artificial *de novo* exonic splicing enhancers or suppressors (Figure S5A), and indeed the *unc-13* “macroexon” splicing reporter exhibits similar splicing patterns to the wild-type reporter, with many neurons expressing the included isoform and many other neurons expressing the skipped isoform (Figures 6C–6E). On the other hand, the macroexon splicing reporter is completely unaffected by loss of PRP-40, in striking contrast to the wild-type microexon splicing reporter, which is completely dependent on PRP-40 (Figures 6D and 6E). RT-PCR confirms this dramatic loss of dependence on PRP-40 (Figure 6F). These results indicate that manipulating exon size alone, irrespective of any surrounding sequence elements, can determine an exon’s dependence on PRP-40.

We noted that the only two PRP-40-independent microexons we detected are both surrounded by unusually short flanking introns, as small as 49 nt (Figure 6G). Across all small exons (≤51 nt) we observed a strong relationship between flanking intron size and degree of dependence on PRP-40 (Figure 6H). This led us to hypothesize that microexons require PRP-40 unless flanked by very short introns. To test this, we again turned to *unc-13*, which harbors a highly conserved microexon flanked by long introns in both *C. elegans* and related species (Figure S5B). We manipulated the *unc-13* microexon reporter so that the flanking introns were replaced by the small (58 nt) introns flanking the PRP-40-independent *tcer-1* microexon (Figure 6I). Replacing the *unc-13* upstream intron with the *tcer-1* upstream intron results in little change to the *unc-13* splicing pattern in wild-type animals (Figure 6I). Remarkably, however, the microexon is no longer susceptible to loss of PRP-40 (Figure 6I; Figures S5C and S5D). This supports the hypothesis that PRP-40 is required for inclusion of microexons unless flanked by unusually short introns. We were unable to test the *unc-13* downstream intron, as replacing it with the *tcer-1* downstream intron disrupted microexon splicing even in wild-type animals (Figure S5E), suggesting there are important cell-specific *cis*-regulatory elements in the *unc-13* downstream intron.

Together these results indicate that PRP-40-regulated microexons are co-regulated by a substantial number of sequence-specific RNA binding proteins, with no single RNA binding protein playing a clear dominant role. Microexons require PRP-40 specifically because of their small size, and the requirement for PRP-40 is alleviated when a microexon is flanked by small introns.

### Mammalian PRPF40A regulates microexon splicing

**Figure 7.**
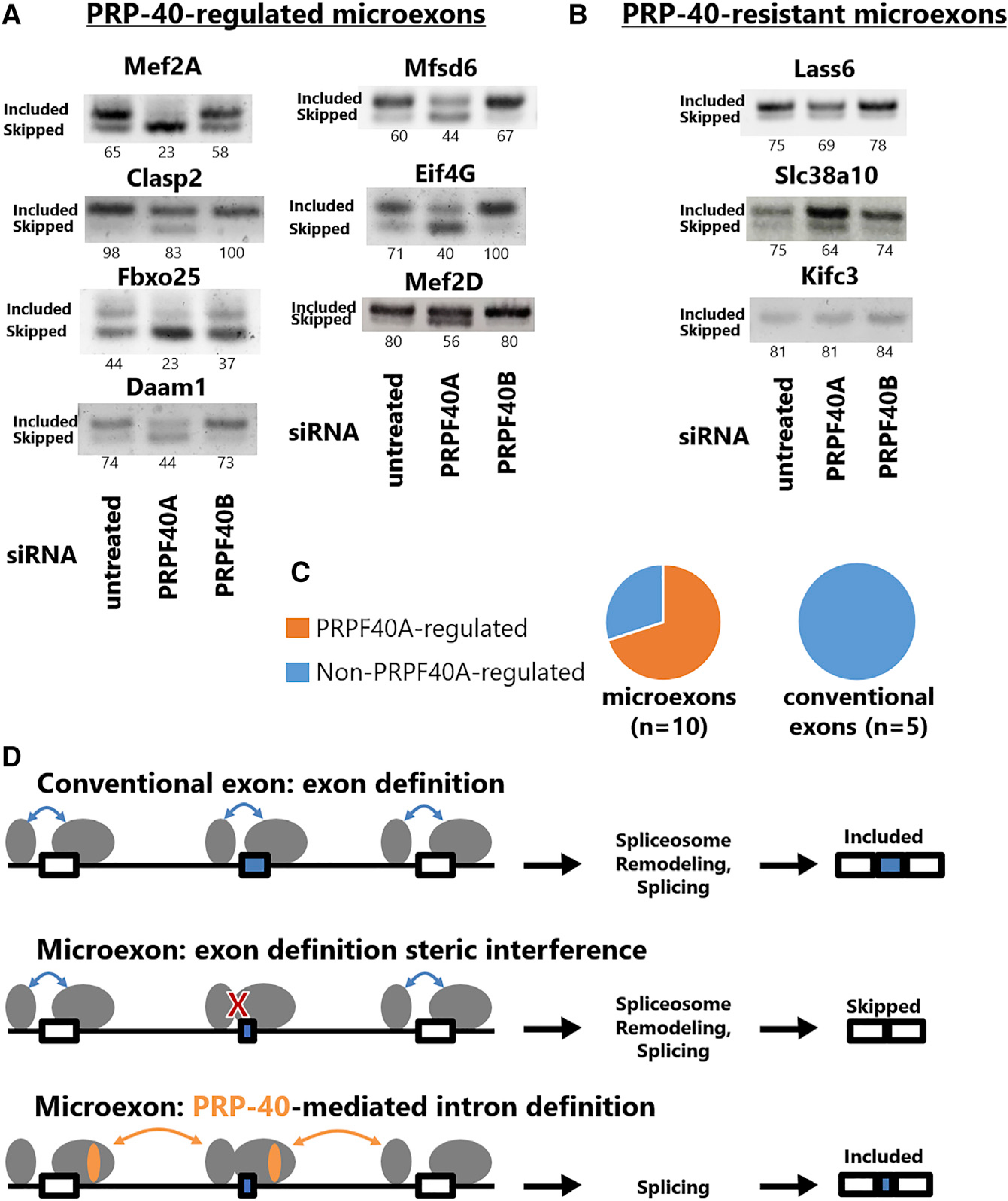
Mammalian PRPF40A regulates microexon splicing (A) PRPF40A siRNA knockdown caused increased skipping in seven of ten of microexons tested, detected by RT-PCR. (B) Three of ten microexons did not undergo appreciable increase in skipping. (C) Summary of RT-PCRs indicating fraction of microexons or conventionally sized exons regulated by PRPF40A. (D) Model for PRP-40-mediated regulation of microexons. For conventionally sized exons, spliceosomal components initially assemble and interact across an exon via exon definition, before subsequently remodeling to splice across the intron. Exon definition is sterically infeasible across microexons because of the prohibitively small sequence causing steric interference between spliceosomal components. Instead, PRP-40 facilitates intron definition across long introns through its protein-protein interactions between the U1 snRNP at the 5′ splice site and the BBP and the branchpoint. See also Figure S6.

We next wanted to investigate whether PRP-40 is an evolutionarily conserved regulator of microexons. To determine whether PRP-40-regulated mechanisms govern mammalian microexon splicing, we tested the function of PRP-40 homologs in mouse neuroblastoma N2a cells. Mammals encode two PRP-40 homologs, PRPF40A and PRPF40B (Figure S6A). We hypothesized that PRPF40A was the factor most likely to function similarly to PRP-40, because PRPF40A has higher sequence similarity to PRP-40 (Figure S6A) and because PRPF40A, but not PRPF40B, has been identified as a component of the U1 snRNP (Wahl et al., 2009). We performed small interfering RNA (siRNA) knockdown of PRPF40A or PRPF40B in mouse N2a cells (Figure S6B) and tested a panel of previously identified neuronal microexons (Gonatopoulos-Pournatzis et al., 2018) using RT-PCR. Of the ten microexons we tested, seven (70%) clearly exhibited decreased inclusion upon PRPF40A knockdown but were unaffected by PRPF40B knockdown (Figure 7A). The remaining three microexons exhibited mild or no changes upon PRPF40A knockdown (Figures 7B and 7C), suggesting that they either do not depend on PRPF40A or are not sensitive to partial PRPF40A knockdown. In contrast, we tested five conventionally sized exons (> 51 nt) and found that none was affected by loss of PRPF40A (Figure S6C). Together these results indicate that mammalian PRPF40A is a regulator of microexon splicing and suggest that the role of PRP-40 as a central regulator of microexons is a widespread phenomenon among animal species.

## DISCUSSION

### Regulation of alternative splicing by a core spliceosomal subunit

Alternative splicing is traditionally considered to be regulated by sequence-specific RNA binding proteins that interact with the pre-mRNA and influence spliceosomal assembly (Fu and Ares, 2014). Recently, core components of the spliceosome itself have been shown to play regulatory roles in alternative splicing (Dvinge et al., 2019; Papasaikas et al., 2015; Park et al., 2004; Saltzman et al., 2011). Reduced levels of some spliceosomal proteins or small nuclear RNAs (snRNAs) have been found to preferentially affect specific types of splicing. For example, U1 snRNA knockdown preferentially leads to 5′ splice site dysregulation, while U4 or U6 snRNA knockdown preferentially leads to intron retention (Dvinge et al., 2019). We now extend this concept to show that PRP-40, a core component of the U1 snRNP, is a central regulator of microexons. As in the cases cited above, we find that *prp-40* mutants do not exhibit hallmarks of global splicing inefficiency: only a small fraction of alternative exons are dysregulated, and widespread intron retention is not observed.

Microexon dysregulation is not an inevitable consequence of spliceosome perturbation, as perturbing other spliceosomal components in various organisms, including *C. elegans*, does not result in microexon dysregulation (Dvinge et al., 2019; Mayerle et al., 2019; Zahler et al., 2018). PRP-40, as a central regulator of microexons, is therefore a member of a small but growing group of core spliceosomal components with specific regulatory roles in alternative splicing. We speculate that future studies may identify additional spliceosomal components that regulate other coherent classes of alternative splicing.

### Neuronal microexon networks as a widely conserved phenomenon

Work in vertebrate systems has demonstrated that microexons are enriched in neuronally expressed genes, are preferentially included in the nervous system, and are dysregulated in individuals with autism spectrum disorder (Irimia et al., 2014). We now show that *C. elegans* microexons are likewise highly enriched in genes with neuronal function. Moreover, *prp-40* mutants, in which most microexon inclusion is lost, exhibit neuronal and behavioral defects. The phenotypic consequences of specific dysregulated microexons in *C. elegans* remains to be determined, but in other systems a number of specific microexons have been found to mediate specific neuronal phenotypes (Gonatopoulos-Pournatzis et al., 2020; Johnson et al., 2019; Nakano et al., 2018; Ohnishi et al., 2014; Wang et al., 2015). We expect that similar phenomena will be found to occur in *C. elegans*. Together these results show that the existence of neuronal microexon networks is a common theme across widely divergent metazoan species.

### PRP-40 as a central regulator of microexon splicing

Whereas PRP-40 is required for inclusion of nearly every detectable microexon, we identified a number of sequence-specific splicing factors that regulate small subsets of microexons. *Prp-40* genetically and physically interacts with one of these RNA binding proteins, *exc-7/ELAV*. We speculate that this may represent a common mode of action for PRP-40. If the activity of EXC-7 and PRP-40 in regulation of *unc-16* splicing is generalizable to regulation of microexons, then sequence-specific RNA binding proteins could bind *cis*-elements in the vicinity of microexons, recruit PRP-40, and thus ensure spliceosomal recognition. In principle, some microexon dysregulation in *prp-40* mutants could be an indirect consequence of PRP-40 regulating an intermediary factor that stimulates microexons. However, *prp-40(−)* RNA-seq does not reveal gene expression changes in known microexon regulators, and known microexon regulators affect very small subsets of *prp-40-*regulated microexons in *C. elegans* (Figure 6B; Figure S6D). Coupled with biochemical and genetic work in yeast and mammalian cells (Becerra et al., 2016; Kao and Siliciano, 1996, p. 40; Li et al., 2019), we therefore favor a model in which PRP-40 directly mediates microexon splicing, with regulatory input provided by sequence-specific RNA binding proteins.

According to this model, although PRP-40 expression is ubiquitous, microexon inclusion can be regulated in a developmental or cell-specific manner on the basis of the complement of sequence-specific RNA binding proteins expressed. For example, in the *C. elegans* nervous system, neuronally expressed splicing factors such as *exc-7* and *fox-1* could mediate microexon inclusion by recruiting PRP-40. In other tissues, although PRP-40 is expressed, if its co-activators are absent then PRP-40 will fail to be recruited to microexons, resulting in exon skipping. A similar phenomenon may occur in mammalian cells, in which unbiased proteomics experiments have identified PRPF40A as an interaction partner for a number of microexon-regulating RNA binding proteins (Gonatopoulos-Pournatzis et al., 2018), including the neuron-specific splicing factor Srrm4 (Torres-Méndez et al., 2019).

On the other hand, a recent genome-wide CRISPR-Cas9 screen for microexon regulators in mouse N2a cells did not identify PRPF40A (Gonatopoulos-Pournatzis et al., 2018), while we find that at least one of the microexons screened (Mef2d) is indeed regulated by PRPF40A (Figure 7A). One potential explanation for this difference could be that PRPF40A disruption via CRISPR-Cas9 results in fitness defects leading to cell loss or “dropout” in the CRISPR-Cas9 screen. If so, this would highlight the utility of traditional forward genetic screens in model organisms such as *C. elegans* as a complementary approach for studying splicing regulation. In the future it will be interesting to determine the extent to which specific mechanisms of PRP-40-mediated microexon recognition, and its role as a central coordinator of microexon splicing, are conserved across diverse animal species.

### Does PRP-40 regulate microexon inclusion by facilitating intron definition?

We present a speculative mechanistic model for PRP-40 regulation of microexons (Figure 7D) on the basis of the present study as well as previous structural and functional work on yeast PRP40. In yeast, PRP40 mediates “bridging” interactions across an intron between the 5^0^ splice site (via its FF domains interacting with U1–70K) and the branchpoint sequence (via its WW domains interacting with BBP) (Abovich and Rosbash, 1997; Li et al., 2019). This is an essential element of the “intron definition” mechanism by which yeast introns are recognized (Abovich and Rosbash, 1997; Li et al., 2019).

In contrast, metazoan splicing is believed to occur largely through “exon definition” mechanisms (Fox-Walsh et al., 2005; Osella and Caselle, 2009; Robberson et al., 1990), by which the spliceosome initially recognizes and assembles across an exon before subsequently undergoing rearrangements to enable intron excision. This may be favorable for organisms such as mammals with long introns, in contrast with the relatively short introns in yeast (Ast, 2004). It has been proposed that microexons provide challenges to exon definition due to steric interference or incompatibility between spliceosomal components when in such close proximity (Black, 1991). In such a scenario, microexon splicing might require intron definition (Sterner et al., 1996), and PRP-40 would be a prime candidate for facilitating intron definition across lengthy intronic sequence space through its protein-protein interactions with factors at both the 5′ and 3′ ends of an intron (Abovich and Rosbash, 1997; Li et al., 2019) (Figure 7D).

Our data are consistent with this model: first, across the transcriptome PRP-40 becomes increasingly necessary for inclusion of small exons as the flanking introns become longer. This is consistent with the notion that intron definition is a “default” splicing mechanism for short introns (Fox-Walsh et al., 2005) but that intron definition becomes less efficient for long introns, thus necessitating additional regulatory control, provided by PRP-40. Second, we can relieve *unc-13* microexon dependence on PRP-40 by replacing its naturally occurring long upstream intron with an unusually short intron. Together these data support the model that exons below a certain size require PRP-40 to facilitate exon inclusion via spliceosomal intron definition. This model of PRP-40 activity suggests a unifying framework for regulation of both microexons and other types of alternative splicing (Figure 3C): PRP-40 is required when exon definition mechanisms are insufficient, whether because of size constrains or because of other constraints (sequence, structure, etc.) Under such conditions, PRP-40 is required for splicing via intron definition.

## STAR⋆METHODS

### RESOURCE AVAILABILITY

#### Lead contact

Requests for reagents and resources should be directed to the lead contact, Adam Norris (adnorris@smu.edu).

#### Materials availability

All *C. elegans* strains generated in this study are freely available upon request.

#### Data and code availability

The RNA-seq datasets generated during this study are available at the NCBI SRA (PRJNA684142). This study did not generate code.

### EXPERIMENTAL MODEL AND SUBJECT DETAILS

#### *C. elegans* strain maintenance

*C. elegans* were maintained by standard techniques (Brenner, 1974) on NGM agar plates seeded with OP50 *E. coli*. *prp-40* mutants were balanced with the hT2 or the eT1 balancer to create stable heterozygous lines. The *csb3* allele (deletion) was used for all assays unless otherwise noted. RNA binding protein deletion mutants were generated using CRISPR/Cas9 and characterized as previously described (Calarco and Norris, 2018; Norris et al., 2015). Strain PHX1811 *zk1098.1(syb1811)* [PRP-40::wrmScarlet] was generated by SunyBiotech. Some strains were provided by the CGC, which is funded by NIH Office of Research Infrastructure Programs (P40 OD010440).

#### N2a cell maintenance

N2a cells were grown in OptiMEM medium plus DMEM, high glucose with L-glutamine, with 5% FBS and penicillin/streptomycin at 37°C and 5% CO_2_. Cells were transfected with 10 nM pools of siRNA (siGENOME, Dharmacon) using RNAiMax, according to manufacturer recommendations (Life Technologies). Cells were harvested 72 hours after transfection for RT-PCR analysis.

### METHOD DETAILS

#### Mutagenesis and mapping

Genetic screen was performed as previously described (Norris et al., 2014). P0 worms expressing the *unc-16* alternative splicing reporter were mutagenized with 47 mM ethyl methanesulfonate (EMS) for four hours. Single F1s were sorted into individual wells of a 96-well plate by a Union Biometrica COPAS worm sorter. An estimated ~7,000 haploid genomes were screened in total. The *unc-16* alternative splicing reporter in pooled F2-F3 mutant worms was analyzed on a Zeiss Axioskop 2 compound fluorescent microscope, and the splicing pattern in the ventral nerve cord was the primary phenotype under consideration. Wells containing lethal mutants were recovered and viable heterozygotes were maintained. To identify causative mutations in lethal strains, we subjected individual 4X-outcrossed homozygous mutants to whole genome amplification and sequencing (Spits et al., 2006). Mapping was performed with the software MAQgene according to previously described protocols (Bigelow et al., 2009).

#### RNA-seq analysis

RNA-seq was performed on stage-matched L3 wild-type or *prp-40(csb3)* animals in biological triplicates. Each biological replicate consisted of 400–500 worms which were hand-selected to ensure all mutant animals were homozygous (lacking the GFP+ hT2 balancer). RNA was extracted using Zymo Direct-Zol Miniprep kits, and RNA-seq libraries were generated using NEBNext library prep kits. Paired-end 150 bp sequencing mapped with STAR (Dobin et al., 2013) yielded 35–100 million uniquely mapped reads for each sample. The sequencing data generated during this study are available at the NCBI SRA archive (PRJNA684142). Alternative splicing was analyzed using JUM (Wang and Rio, 2018) requiring exon junctions to be represented by at least 5 junction-spanning reads in at least 2 out of 3 replicates to be considered bona fide splicing events. Significance values were set at FDR-corrected q < 0.01 with a |ΔPSI| ≥ 20. Gene models to determine whether microexons were previously annotated were taken from WBcel235 via NCBI RefSeq.

#### Gene Ontology and MEME analysis

Gene Ontology analysis was performed on genes containing PRP-40-regulated microexons (microexons undergoing |ΔPSI| ≥ 20, q < 0.01) versus genes lacking such microexons, with g:Profiler (Raudvere et al., 2019), querying either Biological Processes or Cellular Components. Ontology networks were visualized using Cytoscape’s Enrichment Map plugin (Shannon et al., 2003) with a node cutoff of FDR < 0.05. Cluster names were generated with AutoAnnotate MCL using similarity coefficients and WordCloud “biggest words” on clusters of at least 5 nodes. Analysis of Motif Enrichment was performed using MEME suite 5.1.1 (Bailey et al., 2009) (CIS-BP RNA *C. elegans*). Upstream and downstream introns flanking microexons strongly regulated by PRP-40 (> 22 ΔPSI) were compared against a corresponding background of those not regulated by PRP-40 (< 8 ΔPSI).

#### Co-immunoprecipitation

The samples were prepared by mechanically homogenizing the worm bed in homogenization buffer (15mM HEPES-NaOH pH 7.4, 1.5 mM MgCl_2_, 10 mM KCl, 0.1 mM EDTA, 0.5 mM EGTA) supplemented with protease inhibitors (Roche) followed by homogenization by sonicator. The samples were incubated with rabbit Anti-GFP (1:50) (Chromotek) overnight at 4°C. The antigen-antibody complex was incubated with proteinA/G beads (Santacruz) for 3–4 hours at 4°C. The immunoblots were probed with Rabbit Anti-GFP or mouse monoclonal Anti-mscarlet (Chromotek) with a dilution of 1:1000.

#### Image acquisition and immunohistochemistry

Worms were anesthetized with 30 mM sodium azide and mounted on 2% agarose pad. The images were acquired either on Zeiss LSM 5 confocal or with Zeiss epifluorescence microscope equipped with CCD camera at 60 or 20 X. For intensity analysis of different genotypes, unsaturated images were acquired with constant exposure and gain across the genotypes, and images were analyzed by ImageJ (NIH).

For immunohistochemistry worms were fixed with 2% paraformaldehyde and processed further as described previously (Kumar et al., 2010). In double labeling of nuclear proteins, Rabbit Anti-GFP and rat Anti-mscarlet (Chromotek) antibodies were used with a dilution of 1:500. Respective secondary antibodies were used at a dilution of 1:250 (Chromotek; Jackson laboratories). Images of immunostained worms were acquired under confocal microscope in sequential mode.

#### Behavioral and pharmacological assays

L3 stage animals of respective genotypes were transferred on M9 buffer droplet (30 ul) on an unseeded plate and the number of thrashes were counted for 30 s.

For aldicarb assay, 0.2mM aldicarb NGM plates were prepared as described earlier (Mahoney et al., 2006). Animals (L3 stage animals) of respective genotypes were incubated on the plate. The paralysis was scored at 30 minutes interval by touching at the nose of worm by platinum wire.

### QUANTIFICATION AND STATISTICAL ANALYSIS

For box-and-whiskers plots, whiskers represent 5th and 95th percentiles, with individual data points beyond these percentiles shown.

In splicing analysis using JUM, significance values were set at FDR-corrected q < 0.01 with a |ΔPSI| of ≥ 20. Single-isoform PSI graphs are represented as median PSI value ± SEM.

Statistical tests for behavioral analyses were paired t test with unequal variance and one-way ANOVA. Tests for rescue of *prp-40* lethality and fertility were unpaired two-tailed t test. Significance cutoff of p < 0.01. Analyses were performed in MS excel or Graph-Pad Prism.

## Supplementary Material

1

2

## Figures and Tables

**KEY RESOURCES TABLE:** 

REAGENT or RESOURCE	SOURCE	IDENTIFIER

Antibodies		

Rabbit Polyclonal Anti-GFP	*chromotek*	PABG-1 RRID:AB_2749857
Rat monoclonal anti-RFP	*chromotek*	5F8 RRID:AB_2336064
Mouse monoclonal anti-RFP	*chromotek*	6G6 RRID:AB_2631395
Mouse monoclonal anti-GFP	*chromtek*	3H9 RRID:AB_10773374

Deposited data		

wild-type and *prp-40(csb3)* polyA RNA-Seq (*C. elegans*)	NCBI SRA	PRJNA684142

Experimental models: Cell lines		

N2A (Neuro-2a)	ATCC	CCL-131

Experimental models: Organisms/strains		

exc-7(ot970[exc-7::gfp]) II	CGC, University of Minnesota	OH16020
Ex[unc-13 splicing reporter]	This study	ADN690
Ex[unc-13 macroexon splicing reporter]	This study	ADN692
Ex[unc-13 tcer-1 upstream intron]	This study	ADN827
*zuIs178*	Caenorhabditis Genetics Center	WBTransgene00005242
*vsIs48*	Caenorhabditis Genetics Center	WBTransgene00004893
*juIs73*	Caenorhabditis Genetics Center	WBTransgene00000718
*otIs45*	Caenorhabditis Genetics Center	WBTransgene00001578
Ex[unc-13 tcer-1 downstream intron]	This study	ADN799
Is[unc-16 splicing reporter]	Norris Lab, SMU	ADN630
Is[sad-1 splicing reporter]	Norris Lab, SMU	ADN670
*prp-40(csb3)/hT2*	This study	ADN742
endogenous PRP-40::wrmScarlet	This study	PHX1811
*prp-40(csb5)/hT2*	This study	ADN775
unc-75(e950)	CGC, University of Minnesota	CB950
exc-7(rh252)	CGC, University of Minnesota	NJ683

Oligonucleotides		

unc-16F AAAGTGGAGGACCCAGTACC	This study	N/A
unc-16R CCTCGCCCTTGCTCACTGC		N/A
sad-1F AAACGAGGTCCGACAGTTGG	This study	N/A
sad-1R GAGCCGGGCGAGTATTGATTC	This study	N/A
sad-1R CGATGTAA TTCCTGTTCCACTTCC	This study	N/A
unc-13F AACGAAGACAAGTATTCCACT	This study	N/A
unc-13R ATTCGCCAGACCTGCTT	This study	N/A
unc-13R transgene only AACT TGTGGCCGTTTACGT	This study	N/A
Mef2aF GTGGCTTGAGAACTGCTTATC	This study	N/A
Mef2aR TGAACAGTCGGAAACCAGATC	This study	N/A
Mfsd6 F CTCCTCGCC TTGGGTGACCTTTG	This study	N/A
Mfsd6 R GGCTTCC CGATTCTCACTGGTCC	This study	N/A
Clasp2 F TGGAGGAGGCAGTAGCTGATG	This study	N/A
Clasp2 R AGCGTTCTGAACACGCACTAG	This study	N/A
Eif4g1F ACAAATGAACACGCCTTCTC	This study	N/A
Eif4g1R GGCCCGGCTAGGGTAGAAGT	This study	N/A
Fbxo25F CTACACTTCTGCCGCCACTG	This study	N/A
Fbxo25R AGACACAGGAGTGAAGCAGC	This study	N/A
Mef2d F ACAAAGTC ATCCCTGCCAAGTCTC	This study	N/A
Mef2d R GAGTAAACTTG GTGTTGCCACGGA	This study	N/A
Daam1 F CCTGAAGAC CTAGAAAGAACGTTC	This study	N/A
Daam1 R GAAGGATGTT GCAATTCTGAGCTC	This study	N/A
Lass6 F TCCTGGTGGGTTTTTAACCTGCT	This study	N/A
Lass6 R TGGTTCCGTT GGTGGTTGTTGAAG	This study	N/A
Slc38a10 F GGAGAAGAA GGAGGCTGAGCA	This study	N/A
Slc38a10 R CTGCTGCT CTTGGATCACCTG	This study	N/A
Kif3c F GTAGCCGCCCAGGTTCCATCC	This study	N/A
Kif3c R GGCAGCGTGGCACATCTTCAC	This study	N/A
prpf40aF CAATAGAACTGGATGCTGTCTG	This study	N/A
prpf40aR CTCAAACGCTGGTTCCTTTAC	This study	N/A
prpf40bF ATGATGTCCTCTTCTTCCTGG	This study	N/A
prpf40bR CACTGCTCATCCCATCCAGG	This study	N/A
Gapdh F AGGCCGGTGCTGAGTATGTC	This study	N/A
Gapdh R TGCCTGCTTCACCACCTTCT	This study	N/A
Numa1 F GGAGGTGATG ACTGCCAAGTACG	This study	N/A
Numa1 R GCTGCACCTTGCTGGCTTGG	This study	N/A
FN1F TGGGTGTCACCTGACTGAAC	This study	N/A
FN1R AGAACCGGAACGGAGAAAGC	This study	N/A
NCAM1F TTTGTTTGTGTGGCATCGTTGG	This study	N/A
NCAM1R AAAGAACCCATTGTGGAGGTC	This study	N/A
NR1F CCTACAAGCGACACAAGGATG	This study	N/A
NR1R AGCAGCAGGACTCATCAGTG	This study	N/A
Itga6F ATCCTCCTGGCTGTTCTTGCC	This study	N/A
Itga6R TCCGCACCGCATGGTATCGG	This study	N/A
siGENOME Mouse Prpf40a SMARTpool siRNA	Dharmacon	56194
siGENOME Mouse Prpf40b SMARTpool siRNA	Dharmacon	54614
siGENOME Mouse Srrm4 SMARTpool siRNA	Dharmacon	68955

Software and algorithms		

STAR	Dobin et al., 2013. Bioinformatics	https://github.com/alexdobin/STAR
JUM	Wang and Rio, 2018 PNAS	https://github.com/qqwang-berkeley/JUM
Samtools	Li et al., 2009	http://samtools.sourceforge.net/
DESeq2	Love et al., 2014	N/A
ImageJ		https://imagej.nih.gov/ij/

## Highlights

- U1 spliceosomal component PRP-40 regulates alternative splicing
- PRP-40 is required for microexon inclusion in *C. elegans* and mammalian cells
- Increased exon size or decreased flanking intron size rescues PRP-40 dependence
- PRP-40 may direct intron definition in cases in which exon definition is impossible
