# Supplementary material for: Spliceosomal component PRP-40 is a central regulator of microexon splicing
Source: Cell Rep. Author manuscript; Available in PMC 2021 Aug 20. (PMC8378409; doi:10.1016/j.celrep.2021.109464)
Supplement: 1 [file NIHMS1730369-supplement-1.pdf]

Cell Reports, Volume *36*

## Supplemental information

# Spliceosomal component PRP-40 is a central regulator of microexon splicing

**Bikash Choudhary, Olivia Marx, and Adam D. Norris**

## SUPPLEMENTAL FIGURES

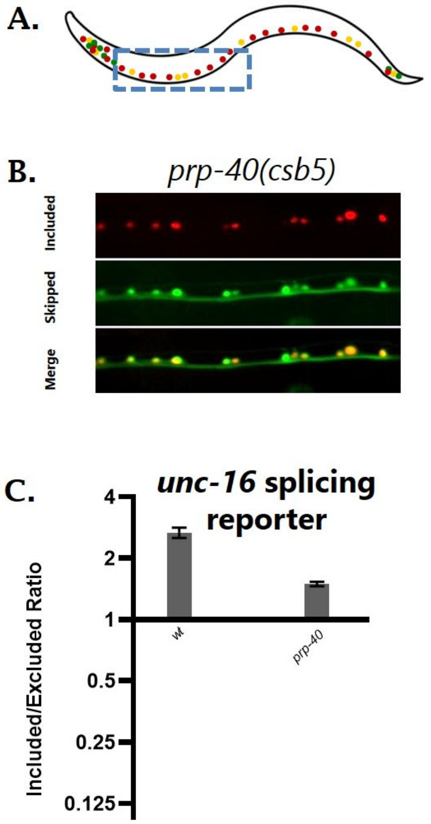


**Figure S1: Additional *prp-40* mutant phenotype.** Related to Figure 1. (A) Cartoon of worm indicating the ventral nerve cord region imaged in C. (B) *prp-40(csb5)* premature stop codon mutations and *prp-40(csb3)* deletion (See Fig 1) have identical phenotypes with regard to *unc-16* splicing: all motor neurons express both the included and the skipped isoforms. p*rp-40(csb3)* is used henceforth as a reference allele. (C) Fluorescence quantitation of *unc-16* splicing reporter GFP (excluded isoform) / RFP (included isoform) in excitatory motor neurons.

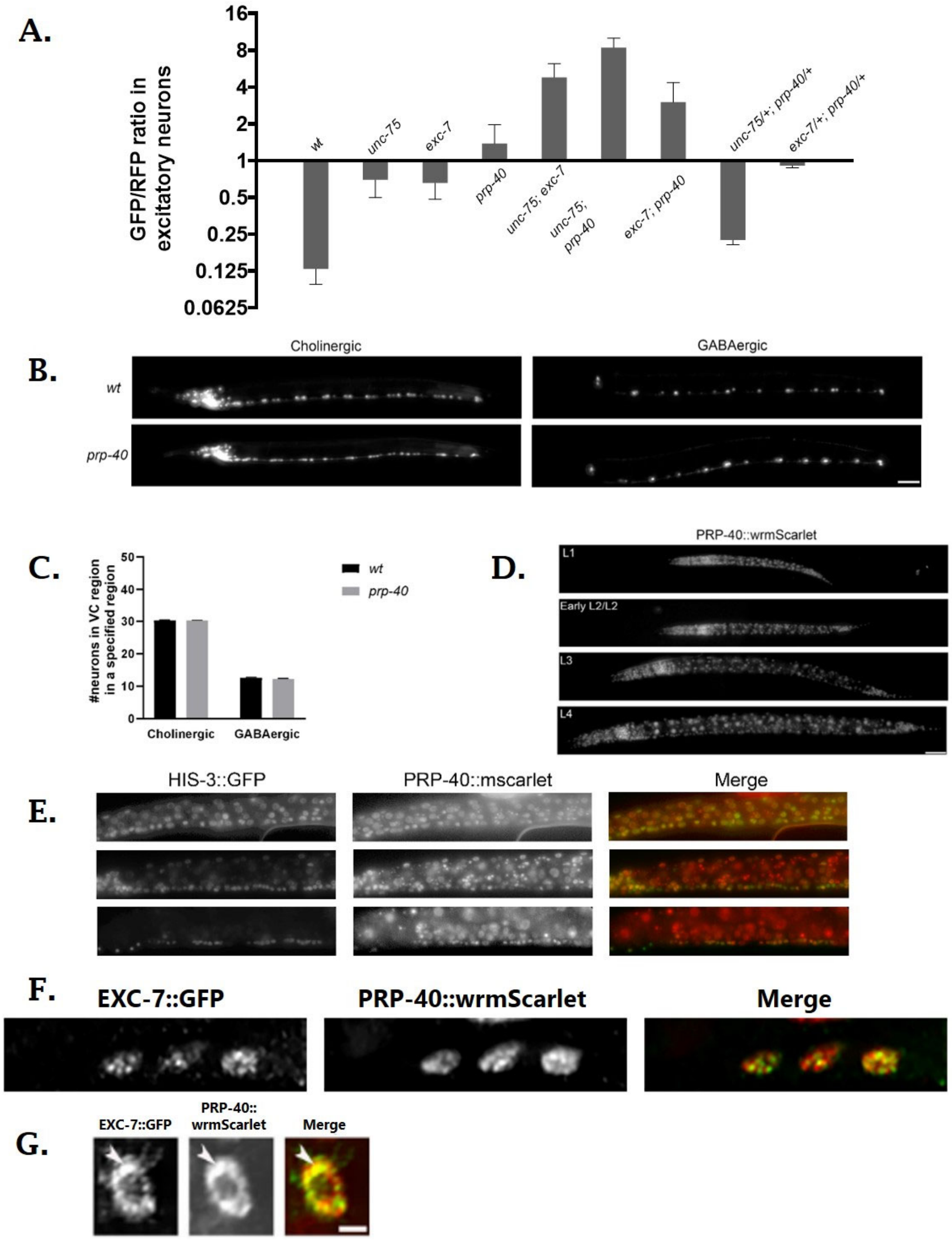


**Figure S2: Expression of PRP-40 and EXC-7.** Related to Figure 2. (A) Fluorescence quantitation of skipped (GFP) / included (RFP) *unc-16* isoform in excitatory cholinergic motor neurons. (B) Cell identity markers for both Cholinergic (*unc*-17p) and GABAergic neurons (*unc-25p*) of the ventral nerve cord reveal that *prp-40* does not disturb the cell identity, number, or position of motor neurons. (C) Quantification of cell numbers within the ventral nerve cord likewise show no difference between wild-type and *prp-40* mutants. (D) PRP-40 expression is widespread throughout development, and not subject to any obvious developmental regulation. (E) PRP-40 is ubiquitously expressed in nuclei, as visualized by histone HIS-33::GFP (A) EXC-7::GFP and PRP-40::wrmScarlet co-localize in the nucleus of ventral cord motor neurons. (F) PRP-40 is expressed ubiquitously and localized to the nucleus (as co-labeled with Histone HIS-3::GFP). (G) Co-occurrence of EXC-7 and PRP-40 in puncta of muscle nucleus (nucleus is filled with fluorescence, nucleolus is devoid of fluorescence), arrowhead denoting an instance of EXC-7 and PRP-40 co-localization. Scale bar represents 6 µm.

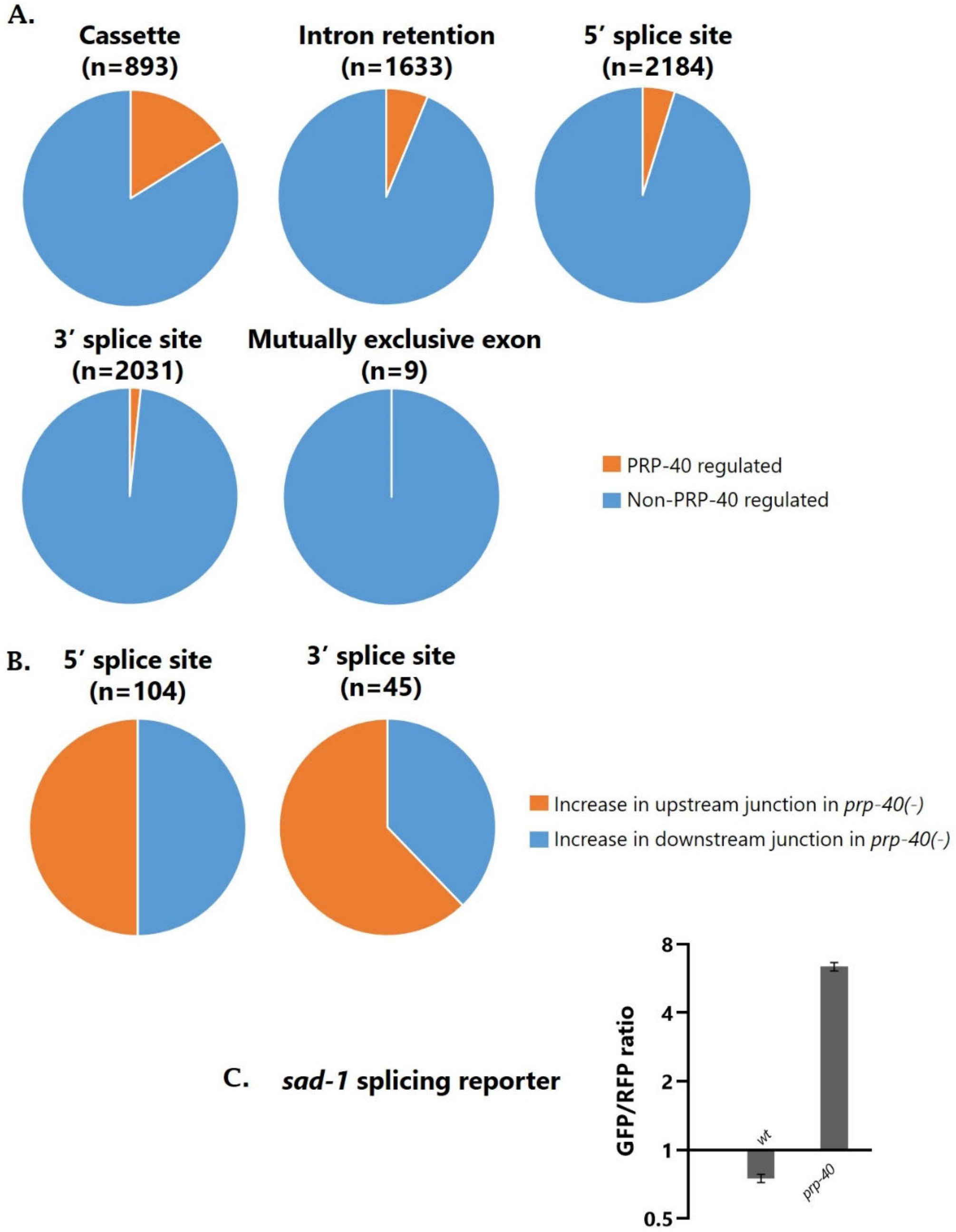


**Figure S3: Classes of alternative splicing affected by *prp-40(-)*.** Related to Figure 3. (A) Fraction of total events detectable that are dysregulated in *prp-40(-)* at ≥ 20% ΔPSI and q≤0.01. (B) Fraction of alternative 5′ and 3′ splice site choices in which *prp-40(-)* causes increased upstream or downstream junction utilization. Total n represents all detectable events of a given type of alternative splicing that reach a threshold of at least 5 junction-spanning reads in at least 2 of 3 biological replicates. (C) Fluorescence quantitation of *sad-1* splicing reporter GFP (included isoform) / RFP (skipped isoform).

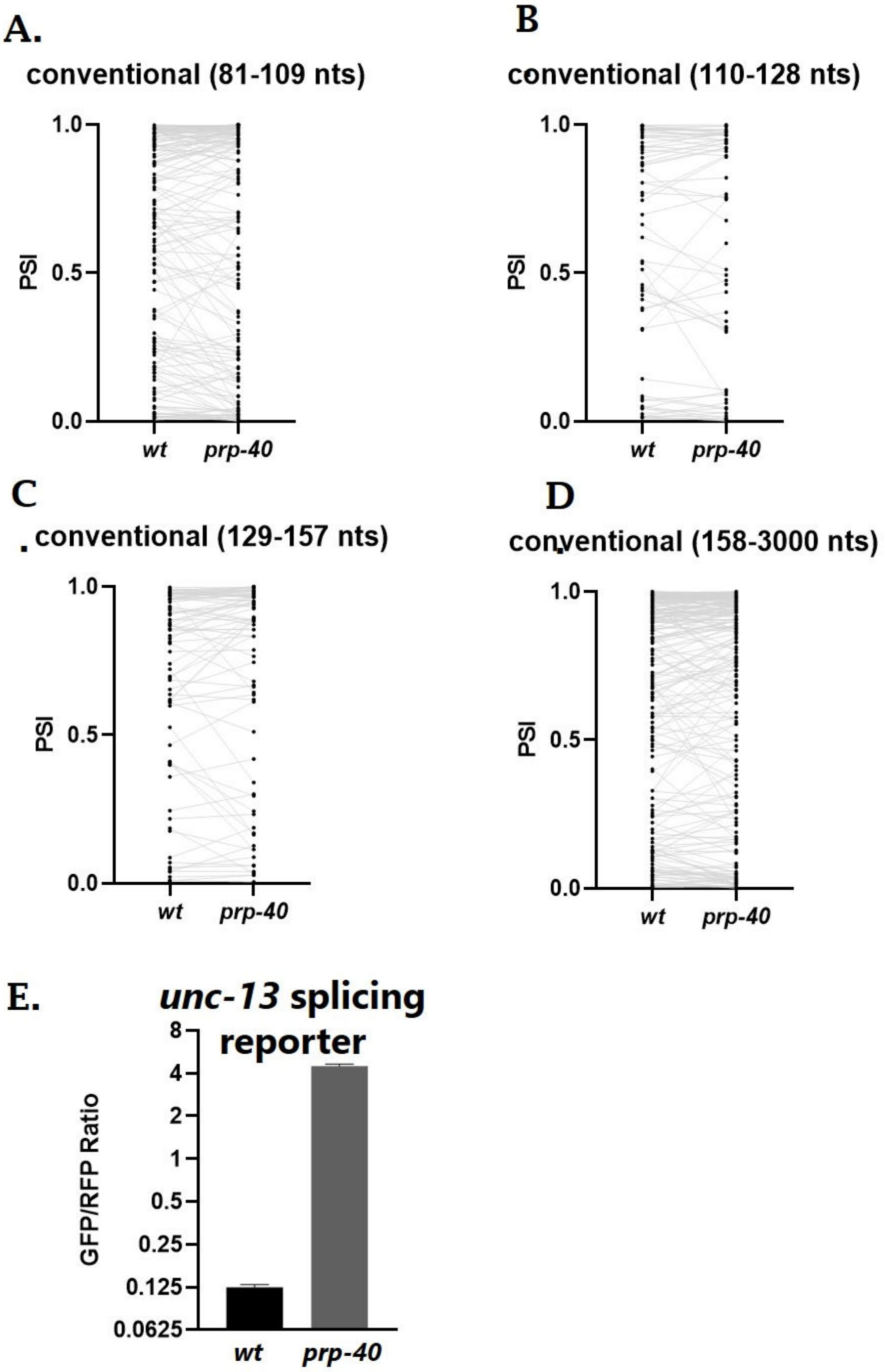


**Figure S4: PRP-40 does not strongly regulate conventionally-sized cassette exons.** Related to Figure 4. (A-D) PSI values for each exon in wild type and in *prp-40(-)* for exons binned by exon size. In contrast to microexons (see Fig 5C), there is no widespread decrease in PSI for conventionally-sized exons in *prp-40(-)*. (E) Fluorescence quantitation of *unc-13* splicing reporter skipped isoform (GFP) / included isoform (RFP).

A.

| Exon | Sequence | AME motifs (E<0.05) |
|---|---|---|
| unc-13 microexon | GTTCTcAAAG | 0 |
| unc-13 “macroexon” | GTTCTAcAAGGTTCTAAAG<br>GTTCTAAAGGTTCTAAAG<br>GTTCTAAAGGTTCTAAAG | 0 |

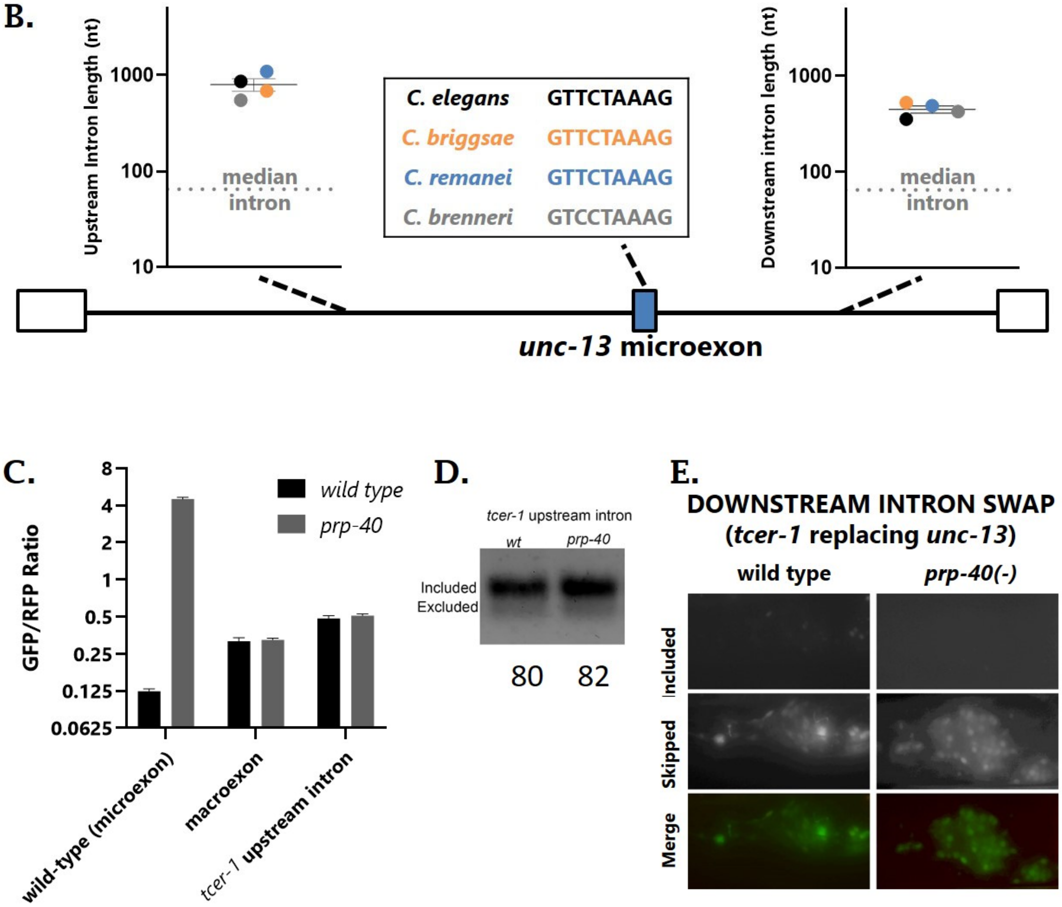


**Figure S5: *unc-13* splicing reporter manipulations.** Related to Figure 6. (A) Conversion of *unc-13* microexon into a “macroexon” by internal duplications does not introduce *de novo* known RNA binding protein *cis*-elements (AME analysis using CIS-BP RNA database). (B) *unc-13* microexon sequences and flanking intron length in four related nematode species, color-coded by species. Included species represent roughly 20 million years of estimated evolutionary distance, comparable to the distance between human and mouse. (C) Quantitation of *unc-13* splicing reporter variants in both wild-type (black bar) and *prp-40* mutants (gray bar). (D) RT-PCR of *unc-13* minigene with upstream intron replaced by *tcer-1* intron reveals that in both wild-type and *prp-40* mutant conditions, the microexon-included isoform is predominant, and no aberrant splicing products are observed. (E) Replacement of downstream intron of *unc-13* with *tcer-1* affects the inclusion pattern in wild-type, and remains the same in the *prp-40(-)* background.

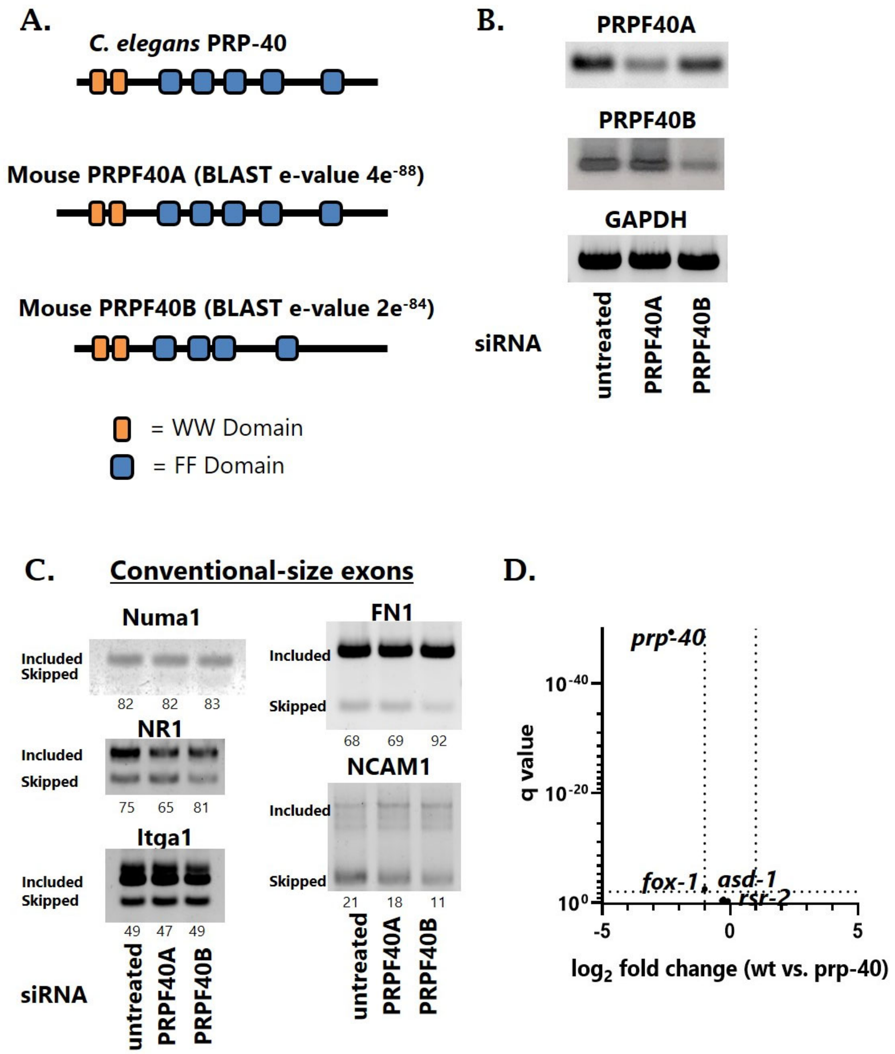


**Figure S6: RT-PCR.** Related to Figure 7. (A) Domain structure and BLAST e-values for *C. elegans* and mouse PRP-40 proteins. (B) RT-PCR or PRPF40A and PRPF40B demonstrating effective siRNA knockdown, in comparison to GAPDH as a negative control. (C) Conventional-sized exons are not affected by loss of PRPF40A or PRPF40B. (D) Gene expression analysis of wild-type vs. *prp-40(-)* using DESeq2. Represented on the volcano plot are known splicing regulators. Only *prp-40* passes statistical cutoffs of q value < 0.01 and $\log_2$ fold change >1. Known microexon regulator homologues (*fox-1* and *asd-1* = Rbfox, *rsr-2* = Srrm2/3/4) fall below these cutoffs.
